# Modeling the Thermodynamic Properties of Saturated Lactones in Nonideal Mixtures with the SAFT-γ Mie Approach

**DOI:** 10.1021/acs.jced.3c00358

**Published:** 2023-11-10

**Authors:** Thomas Bernet, Malak Wehbe, Sara A. Febra, Andrew J. Haslam, Claire S. Adjiman, George Jackson, Amparo Galindo

**Affiliations:** † Department of Chemical Engineering, Sargent Centre for Process Systems Engineering, Institute for Molecular Science and Engineering, Imperial College London, South Kensington Campus, London SW7 2AZ, United Kingdom

## Abstract

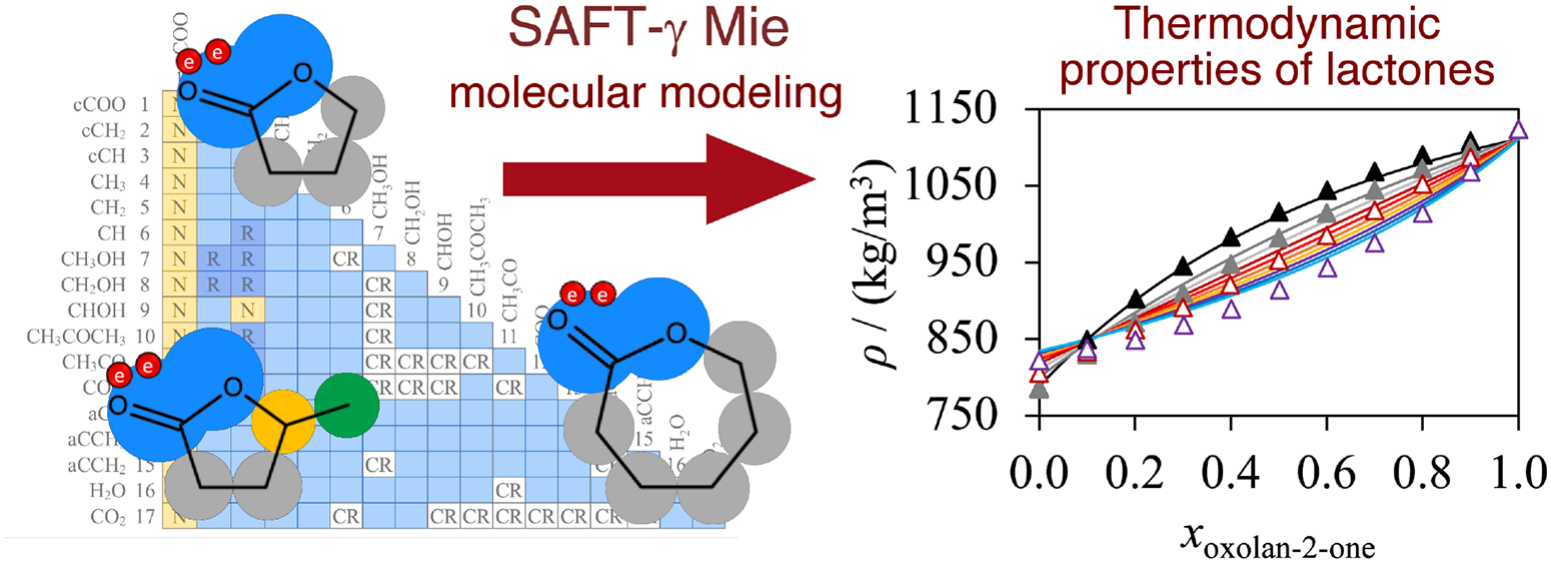

The prediction of the thermodynamic properties of lactones is an important challenge in the flavor, fragrance, and pharmaceutical industries. Here, we develop a predictive model of the phase behavior of binary mixtures of lactones with hydrocarbons, alcohols, ketones, esters, aromatic compounds, water, and carbon dioxide. We extend the group-parameter matrix of the statistical associating fluid theory SAFT-γ Mie group-contribution method by defining a new cyclic ester group, denoted cCOO. The group is composed of two spherical Mie segments and two association electron-donating sites of type e_1_ that can interact with association electron-accepting sites of type H in other molecules. The model parameters of the new cCOO group interactions (1 like interaction and 17 unlike interactions) are characterized to represent target experimental data of physical properties of pure fluids (vapor pressure, single-phase density, and vaporization enthalpy) and mixtures (vapor–liquid equilibria, liquid–liquid equilibria, solid–liquid equilibria, density, and excess enthalpy). The robustness of the model is assessed by comparing theoretical predictions with experimental data, mainly for oxolan-2-one, 5-methyloxolan-2-one, and oxepan-2-one (also referred to as γ-butyrolactone, γ-valerolactone, and ε-caprolactone, respectively). The calculations are found to be in very good quantitative agreement with experiments. The proposed model allows for accurate predictions of the thermodynamic properties and highly nonideal phase behavior of the systems of interest, such as azeotrope compositions. It can be used to support the development of novel molecules and manufacturing processes.

## 1 Introduction

A lactone is an ester in which the functional group −C(=O)–O– is a part of a cycle. The most common approach for the synthesis of lactones involves the intramolecular esterification of hydroxy acids. Numerous other approaches have been developed to obtain specific lactone structures.^1^

Lactones are widely present in fruits, milk, fermentation products, and in many drinks and foods (from plant or animal origin); as such, they are of major interest for the flavor and fragrance industries^1−5^ and are relevant to chemical, biological,^6^ and pharmaceutical processes.^1^ For example, oxolan-2-one is present in a large range of food products^2^ (dehydrated orange powder, tomato, bread, liquid smoke, popcorn, cocoa, coffee, black tea, wines, beef, etc.) but is also used as an organic solvent and as an intermediate in many syntheses^7^ (e.g., for pyrrolidone and derivate compounds).

Knowledge of the thermodynamic properties of lactones can be helpful to synthesize and characterize these molecules and their mixtures and to develop chemical and industrial processes. Oxolan-2-one is, for example, known to be miscible with alcohols, ketones, esters, aromatic compounds, and water, but is not miscible with linear and cyclic aliphatic hydrocarbons.^7^ One of the aims of our work is to study the thermodynamic properties and phase behavior of pure lactones and their binary mixtures with a range of solvents using the SAFT-γ Mie group-contribution approach.^8−11^ Predictive group-contribution approaches can be used in molecular and process design^12−14^ to reduce the number of experiments and material use and to study systems over a large range of thermodynamic conditions.

The statistical associating fluid theory (SAFT)^15,16^ is a molecular equation of state based on statistical physics at the microscopic scale, providing an accurate description of complex fluids over a large range of thermodynamic conditions. In the original approach, molecules were treated as homonuclear chains of tangentially bonded spherical segments with embedded associating sites to mediate directional interactions that mimic hydrogen bonds.^17,18^ More-recent versions have been developed to consider spherical segments interacting through various pair potentials,^19,20^ e.g., the SAFT-VR version for the square-well potential,^21^ soft-SAFT for the Lennard-Jones potential,^22−26^ and SAFT-VR Mie,^27^ which incorporates the Mie potential (a generalized Lennard-Jones potential) and a third-order high-temperature expansion of the attractive contributions to the free energy. A reference chain fluid is used in PC-SAFT,^28^ ePC-SAFT,^29^ and PCP-SAFT.^30^ The PC-SAFT and PCP-SAFT approaches have been used to model 5-methyloxolan-2-one,^31,32^ and the polymerization of oxepan-2-one^33^ and of ω-pentadecalactone^34^ in a mixture of carbon dioxide and dichloromethane have also been studied with PC-SAFT. These approaches, however, are not group-contribution methods, and as a result the molecular parameters presented are not transferable to other lactones or their mixtures.

The SAFT-VR Mie equation was recast as a group-contribution approach in the SAFT-γ Mie equation of state^8−11^ in which molecules are modeled in terms of their constituent chemical moieties (groups) such that once a group is characterized the thermodynamic properties of molecules and mixtures containing the group can be predicted. The method has been shown to deliver an accurate prediction of a broad range of equilibrium thermodynamic properties, including vapor–liquid equilibria (VLE), liquid–liquid equilibria (LLE), and solid–liquid equilibria (SLE), as well as single-phase and derivative properties.^11^ Furthermore, the approach can be used to develop SAFT-γ transferable force-field parameters for use in molecular simulation.^35^ An update of the current capabilities of the SAFT-γ Mie method and the available group interactions can be found in Haslam et al.^11^

Recently, the SAFT-γ Mie approach has also been applied to systems of pharmaceutical interest: octanol–water partition coefficients for a range of active pharmaceutical ingredients were predicted by Hutacharoen et al.,^36,37^ aqueous mixtures of choline and geranate were modeled by Di Lecce et al.,^38^ solubility predictions were obtained for mefenamic acid in a range of solvents by Febra et al.,^39^ and pH solubility profiles of aqueous buffered solutions of ibuprofen and ketoprofen were predicted by Wehbe et al.^40^ It has also been used to develop accurate models of amines and alkanolamines of interest in the field of carbon capture.^41,42^ In addition, the predictive capability of SAFT-γ Mie has been tested with the Clapeyron.jl toolkit by Walker et al.,^43^ and the transferability of the SAFT-γ Mie parameters has been examined by Crespo and Coutinho.^44^ An alternative treatment, referred to as (structural) s-SAFT-γ Mie,^45−47^ has also been proposed to take into account functional group interactions.

Here, we extend the SAFT-γ Mie group-contribution method to represent the family of saturated lactones. The transferability of the new parameters is assessed for a large set of compounds of the lactone family. The parameter estimations and the predictive calculations are carried out by considering vapor-pressure, density, and vaporization-enthalpy data of pure compounds and VLE (including bubble and dew temperature, bubble and dew pressure, and azeotrope composition and temperature), LLE, SLE, density, and molar excess-enthalpy data of binary mixtures.

**Figure 1:**
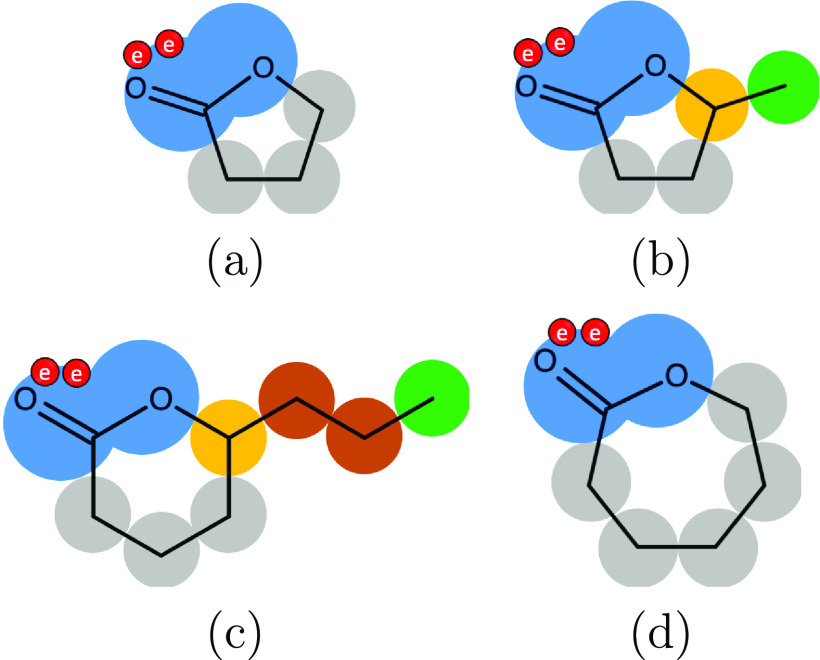
SAFT-γ Mie molecular models of: (a) oxolan-2-one; (b) 5-methyloxolan-2-one; (c) 6-propyloxan-2-one; and (d) oxepan-2-one. The rings of these saturated lactones are modeled with one cCOO group and the corresponding number of cCH_2_ groups (in blue and gray, respectively). The linear side chains are modeled with CH_2_ (in brown) and CH_3_ groups (in green). The carbon participating in the ring and side chain is modeled with a cCH group (in yellow). Association sites are denoted by the smaller red circles, labeled e for electronegative (acceptor) sites.

The different lactones are characterized by their ring size, the presence or absence of side chains, the saturation or unsaturation of the cycle, and the chirality of the atoms of the cycle.^1,3^ Distinct families of lactones can be defined from their structures; for example, phthalides and coumarins contain an aromatic cycle fused to the lactone cycle. The smallest saturated lactone found in nature is oxolan-2-one, which is a five-membered cycle composed of three cyclic methylene (cCH_2_) groups and one lactone (cCOO) group (Figure 1a). The heterocycle results in dipole moments that are larger for lactones than those of free ester chains; for example, the dipole moment of oxolan-2-one is reported as 3.8 D by Longster and Walker^48^ and as 4.27 ± 0.03 D in the CRC Handbook of Chemistry and Physics^49^ compared with 1.9 D for open-chain esters.^48^ In addition, the electron pairs of the oxygen atoms in the lactone group can form hydrogen bonds with hydrogen atoms from other molecules.^50^

In our model, different ring sizes and side chains are accounted for. Chirality, however, cannot be considered with a first-order group-contribution proposition because proximity effects are not taken into account.^11^ As chiral molecules present near identical thermodynamic properties, there is no need to differentiate between these types of isomers.

In Section 2, we present the SAFT-γ Mie approach and parameter-estimation methods. We detail the models and results for saturated lactones in Section 3 by considering pure fluids (Section 3.1.1) and binary mixtures in a range of solvents: saturated hydrocarbons (Section 3.1.2), primary and secondary alcohols (Section 3.2), 2-ketones (Section 3.3), esters (Section 3.4), aromatic compounds (Section 3.5), water (Section 3.6), and carbon dioxide (Section 3.7). We conclude and summarize our main findings in Section 4.

## 2 Methods

### 2.1 SAFT-γ Mie Model and Theory

In the SAFT-γ Mie approach,^8,51^ molecules are modeled as associating heteronuclear chains of fused spherical segments that interact through Mie potentials of variable range, while attractive short-range directional interactions are added by embedding square-well association sites on a given segment. The total Helmholtz free energy *A* of a fluid of nonionic species is expressed as the sum of four contributions:^8−10,51^
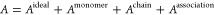
1where *A*^ideal^ is the ideal free energy of the mixture, *A*^monomer^ is the contribution accounting for the Mie segment interactions, *A*^chain^ is the free energy associated with the formation of chains, and *A*^association^ is the contribution to the free energy due to association. (Note that the Born and ionic contributions, which appear in the full SAFT-γ Mie free-energy expression,^11^ are not included in eq 1 since we do not consider any ionic species in our current work.) Detailed expressions for each contribution can be found in previous work.^8−10,51^

Following the group-contribution premise, molecules are modeled in terms of functional groups, and it is assumed that the properties of a given molecule (or mixture) can be obtained by accounting for the appropriate contributions of the groups. The parameters characterizing any given group are treated as transferable to other molecules and mixtures containing the same group. The SAFT-γ Mie representation of four saturated lactones (oxolan-2-one, 5-methyloxolan-2-one, 6-propyloxan-2-one, and oxepan-2-one) can be seen in Figure 1. A given group *k* incorporates a number ν_k_^*^ of identical spherical segments and a shape factor *S*_*k*_ (0 ≤ *S*_*k*_ ≤ 1), which is introduced to characterize the contribution of the segment to the overall molecular properties of the molecule. In the simplest case, the interaction between two segments of groups *k* and *l* is described through a Mie potential:^8^
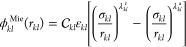
2where *r*_*kl*_ is the distance between the centers of the two segments, σ_*kl*_ is the segment diameter, ε_*kl*_ is the depth of the potential well, and λ_*kl*_^r^ and λ_*kl*_^a^ are the repulsive and attractive exponents of the Mie potential, respectively. The prefactor  is a function of the exponents that ensures that the minimum of potential is −ε_*kl*_, and can be expressed as
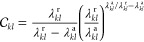
3When relevant, hydrogen bonding and strong polar interactions are represented by short-ranged square-well association sites placed on the segments.^52^ The parameter *N*_ST,*k*_ corresponds to the number of association site types in a group *k*, and *n*_*k*,*a*_ corresponds to the number of sites of a given type *a* = 1, ..., *N*_ST,*k*_. The interaction between a site of type *a* placed on a segment of type *k* and a site of type *b* placed on a segment of type *l* is given by
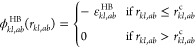
4where *r*_*kl*,*ab*_ is the distance between the centers of the two sites, ε_*kl*,*ab*_^HB^ is the association energy, and *r*_*kl*,*ab*_^c^ is the cutoff range of the interaction. Site *a* is positioned at distance *r*_*kk*,*aa*_^d^ from the center of segment *k*, and site *b* is positioned at distance *r*_*ll*,*bb*_^d^ from the center of segment *l*. The range of the association can equivalently be described by the cutoff *r*_*kl*,*ab*_^c^ or by the bonding volume *K*_*kl*,*ab*_^HB^ for given values of *r*_*kk*,*aa*_^d^ and *r*_*ll*,*bb*_^d^. The interactions between groups *k* and *l* involve unlike parameters, which can be obtained through combining rules^8^ (CR) in the first instance. In our current work, however, ε_*kl*_, *K*_*kl*,*ab*_^HB^, and ε_*kl*,*ab*_^HB^ are systematically estimated by comparing target calculated and experimental thermophysical properties of pure fluids or mixtures in which the functional group is present, and λ_*kl*_^*r*^ is occasionally estimated for better agreement.

The equilibrium thermodynamic properties of the fluid with *c* components can be determined from the total Helmholtz free energy *A* at a temperature *T*, volume *V*, and vector number *N* of molecules, composed of elements *N*_*i*_ of compounds *i*, such that  is the total number of molecules in the system. The pressure is obtained from
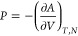
5and the chemical potential of a compound *i* is obtained from
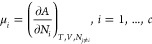
6These standard relations can be used to determine the fluid-phase equilibrium conditions.

The solid–liquid solubility of compound *i* in a given solvent at a given temperature *T* and pressure *P* is obtained by solving the equality between the chemical potential of *i* in the solid phase, assumed pure here, and in the liquid phase: μ_*i*_^S^(*T*,*P*,*x*_*i*_^S^=1) = μ_*i*_^sat^(*T*,*P*,**x**^sat^), where **x**^sat^ is the mole fraction of the saturated solution. The solute mole fraction *x*_*i*_^sat^(*T*,*P*,**x**^sat^) can be calculated as^39^
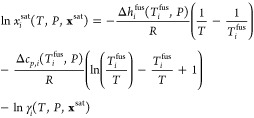
7where *T*_*i*_^fus^ is the melting temperature, Δ*h*_*i*_^fus^(*T*_*i*_^fus^,*P*) is the molar enthalpy of fusion, Δ*c*_*p*,*i*_(*T*_*i*_^fus^,*P*) = *c*_*p*,*i*_^L^(*T*_*i*_^fus^,*P*) – *c*_*p*,*i*_^S^(*T*_*i*_^fus^,*P*) is the difference at *T*_*i*_^fus^ between the molar heat capacity of the liquid phase *c*_*p*,*i*_^L^(*T*_*i*_^fus^,*P*) and the molar heat capacity of the solid phase *c*_*p*,*i*_^S^(*T*_*i*_^fus^,*P*), and γ_*i*_(*T*, *P*, **x**^sat^) is the activity coefficient calculated with SAFT-γ Mie.

Although neglecting Δ*c*_*p*,*i*_(*T*_*i*_^fus^,*P*) can significantly affect the solubility prediction,^53−55^ depending on the mixture and the temperature, experimental values for Δ*c*_*p*,*i*_(*T*_*i*_^fus^,*P*) are unknown for most of the molecules considered in our work. Hence, we do not take this contribution into account in our current calculations. Thus, eq 7 is approximated as
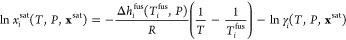
8The molar enthalpies of fusion Δ*h*_*i*_^fus^(*T*_*i*_^fus^,*P*) and melting temperatures *T*_*i*_^fus^ of the lactones and solvents studied in the current work are reported in Table 1, together with their corresponding sources.^56−63^

**Table 1:** Molar Enthalpy of Fusion Δ*h*_*i*_^fus^ and Melting Temperature *T*_*i*_^fus^ of Lactones and Solvents Used in All of the SAFT-γ Mie Calculations and Predictions (cf. eq 8) in Our Current Work

compound *i*	Δ*h*_*i*_^fus^(*T*_*i*_^fus^, *P* = 1 bar)/(kJ/mol)	*T*_*i*_^fus^/K	ref
oxolan-2-one	9.57	229.78	(56)
oxepan-2-one	13.82	272.13	(57)
5-methyloxolan-2-one	7.1	238.34	(58)
cyclohexane	2.63	279.75	(56)
methanol	3.21	175.49	(59)
ethanol	5.02	158.65	(56)
propan-1-ol	5.20	147.05	(56)
butan-1-ol	9.28	183.35	(56)
pentan-1-ol	9.83	194.25	(56)
acetone	5.69	178.35	(56)
butan-2-one	8.44	186.48	(56)
pentan-2-one	10.63	196.31	(60)
hexan-2-one	14.9	216.25	(56)
methyl acetate	7.97	175	(56)
ethyl acetate	10.48	189.55	(56)
propyl acetate	11.2	178	(56)
butyl acetate	14.4	199.7	(56)
diethylbutanedioate	−	252.55	(61)
benzene	9.82	278.33	(62)
toluene	6.6	178.2	(63)
ethylbenzene	9.2	178.2	(63)
water	6.0	273.15	(63)
carbon dioxide	7.9	216.6	(63)

### 2.2 Parameter Estimation

The parameters characterizing the groups are determined by minimizing the objective function *f*_obj_:
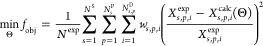
9where Θ is the vector of model parameters, *N*^exp^ is the total number of experimental points considered in the parameter estimation, *N*^S^ is the number of systems (pure compounds/mixtures) used in the estimation, *N*_*s*_^P^ is the number of property types for system *s*, *N*_*s*,*p*_^D^ is the number of experimental data points for system *s* and property *p*, and *w*_*s*,*p*,*i*_ is a weight that is used to control the relative importance of data point *i* for property *p* of system *s*. We consider here the same weight for each point (i.e., *w*_*s*,*p*,*i*_ = 1, for all points). *X*_*s*,*p*,*i*_^exp^ is the *i*^th^ measured value of property *p* of system *s*, and *X*_*s*,*p*,*i*_^calc^(Θ) is the corresponding value calculated with SAFT-γ Mie and the parameters Θ.

The percentage absolute average deviation (%AAD) of a property *p* for a system *s*,
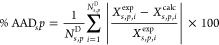
10and the absolute average deviation,
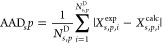
11are used as measures of the accuracy of the theoretical approach.

We use the gPROMS^64^ tools to perform the parameter estimation and calculations. The open-source toolkits SGTPy^65^ and Clapeyron.jl^43^ can also be used to reproduce the calculations.

## 3 Results and Discussion

### 3.1 SAFT-γ Mie Modeling of Lactones: cCOO–cCOO, cCOO–cCH_2_, cCOO–cCH, cCOO–CH_3_, and cCOO–CH_2_ Interactions

#### 3.1.1 Pure Lactones

The lactones considered in our current work are shown in Table 2 together with the details of the groups used in the SAFT-γ Mie modeling.

**Table 2:** List of the Saturated Lactones and Solvents Considered in Our Work, Together with the SAFT-γ Mie Group Representation

compound name	CAS number	molecular formula	SAFT-γ Mie modeling
oxolan-2-one/γ-butyrolactone	96-48-0	C_4_H_6_O_2_	cCOO, cCH_2_ (×3)
oxan-2-one/δ-valerolactone	542-28-9	C_5_H_8_O_2_	cCOO, cCH_2_ (×4)
oxepan-2-one/ε-caprolactone	502-44-3	C_6_H_10_O_2_	cCOO, cCH_2_ (×5)
oxocan-2-one	539-87-7	C_7_H_12_O_2_	cCOO, cCH_2_ (×6)
oxonan-2-one	5698-29-3	C_8_H_14_O_2_	cCOO, cCH_2_ (×7)
oxecan-2-one	6008-27-1	C_9_H_16_O_2_	cCOO, cCH_2_ (×8)
5-methyloxolan-2-one/γ-valerolactone	108-29-2	C_5_H_8_O_2_	cCOO, cCH_2_ (×2), cCH, CH_3_
5-ethyloxolan-2-one/γ-caprolactone	695-06-7	C_6_H_10_O_2_	cCOO, cCH_2_ (×2), cCH, CH_2_, CH_3_
5-propyloxolan-2-one/γ-heptalactone	105-21-5	C_7_H_12_O_2_	cCOO, cCH_2_ (×2), cCH, CH_2_ (×2), CH_3_
5-butyloxolan-2-one/γ-octalactone	104-50-7	C_8_H_14_O_2_	cCOO, cCH_2_ (×2), cCH, CH_2_ (×3), CH_3_
5-pentyloxolan-2-one/γ-nonalactone	104-61-0	C_9_H_16_O_2_	cCOO, cCH_2_ (×2), cCH, CH_2_ (×4), CH_3_
5-hexyloxolan-2-one/γ-decalactone	706-14-9	C_10_H_18_O_2_	cCOO, cCH_2_ (×2), cCH, CH_2_ (×5), CH_3_
6-methyloxan-2-one/δ-caprolactone	823-22-3	C_6_H_10_O_2_	cCOO, cCH_2_ (×3), cCH, CH_3_
6-ethyloxan-2-one/δ-heptalactone	3301-90-4	C_7_H_12_O_2_	cCOO, cCH_2_ (×3), cCH, CH_2_, CH_3_
6-propyloxan-2-one/δ-octalactone	698-76-0	C_8_H_14_O_2_	cCOO, cCH_2_ (×3), cCH, CH_2_ (×2), CH_3_
6-butyloxan-2-one/δ-nonalactone	3301-94-8	C_9_H_16_O_2_	cCOO, cCH_2_ (×3), cCH, CH_2_ (×3), CH_3_
6-pentyloxan-2-one/δ-decalactone	705-86-2	C_10_H_18_O_2_	cCOO, cCH_2_ (×3), cCH, CH_2_ (×4), CH_3_
cyclohexane	110-82-7	C_6_H_12_	cCH_2_ (×6)
*n*-alkanes (ethane, propane, *n*-butane, etc.)	−	C_*n*_H_2*n*+2_	CH_3_ (×2), CH_2_ [×(*n* – 2)], for *n* ≥ 2
methanol	67-56-1	CH_4_O	CH_3_OH
1-alcohols (ethanol, propan-1-ol, butan-1-ol, etc.)	−	C_*n*_H_2*n*+2_O	CH_3_, CH_2_ [×(*n* – 2)], CH_2_OH, for *n* ≥ 2
1,4-butanediol	110-63-4	C_4_H_10_O_2_	CH_2_ (×2), CH_2_OH (×2)
2-methy-1-propanol	78-83-1	C_4_H_10_O	CH_3_ (×2), CH, CH_2_OH
3-methyl-1-butanol	123-51-3	C_5_H_12_O	CH_3_ (×2), CH_2_, CH, CH_2_OH
2-alcohols (propan-2-ol, butan-2-ol, pentan-2-ol, etc.)	−	C_*n*_H_2*n*+2_O	CH_3_ (×2), CH_2_ [×(*n* – 3)], CHOH, for *n* ≥ 3
acetone	67-64-1	C_3_H_6_O	CH_3_COCH_3_
2-ketones (butanone, pentan-2-one, hexan-2-one, etc.)	−	C_*n*_H_2*n*_O	CH_3_CO, CH_2_ [×(*n* – 3)], CH_3_, for *n* ≥ 4
methyl acetate	79-20-9	C_3_H_6_O_2_	COO, CH_3_ (×2)
ethyl acetate	141-78-6	C_4_H_8_O_2_	COO, CH_2_, CH_3_ (×2)
diethyl butanedioate	123-25-1	C_8_H_14_O_4_	COO (×2), CH_2_ (×4), CH_3_ (×2)
benzene	71-43-2	C_6_H_6_	aCH (×6)
toluene	108-88-3	C_7_H_8_	aCH (×5), aCCH_3_
ethylbenzene	100-41-4	C_8_H_10_	aCH (×5), aCCH_2_, CH_3_
water	7732-18-5	H_2_O	H_2_O
carbon dioxide	124-38-9	CO_2_	CO_2_

We introduce here a new cyclic ester group, denoted as cCOO. Ester groups in linear chains can already be modeled with SAFT-γ Mie using the COO group developed in previous work.^8^ The influence of the heterocycle means, however, that a new cCOO group needs to be characterized for an accurate description of the lactone family. It is worth noting that a cyclic ester group^66^ (labeled cy-COO-C) has been introduced in the Modified UNIFAC^67^ approach. As in the case of the linear COO group, two association sites of type e are included in our new cCOO group; no like association occurs between cCOO groups, as no e–e bonding is allowed, but these electronegative sites can bond to H sites in other molecules.

We refer to molecules composed only of one cCOO group and a number of cCH_2_ groups^51^ as ring lactones: three cCH_2_ groups for oxolan-2-one (Figure 1a), four cCH_2_ groups for oxan-2-one, and five cCH_2_ groups for oxepan-2-one (Figure 1d). In addition, we also consider lactones that incorporate an alkyl side chain next to the cCOO group; the intramolecular esterification of hydroxy acids favors the formation of these branches when the OH group of the hydroxy acid is not terminal. Two examples of lactones with side chains can be seen in Figure 1b,c: 5-methyloxolan-2-one and 6-propyloxan-2-one, respectively. In order to treat these molecules, the junction between the ring and the side chain is modeled with a cCH group.^41^ A methyl side chain is composed of a CH_3_ group^8^ only, and longer linear alkyl chains are modeled by one or several CH_2_ groups,^8^ with a terminal CH_3_ group.

**Figure 2:**
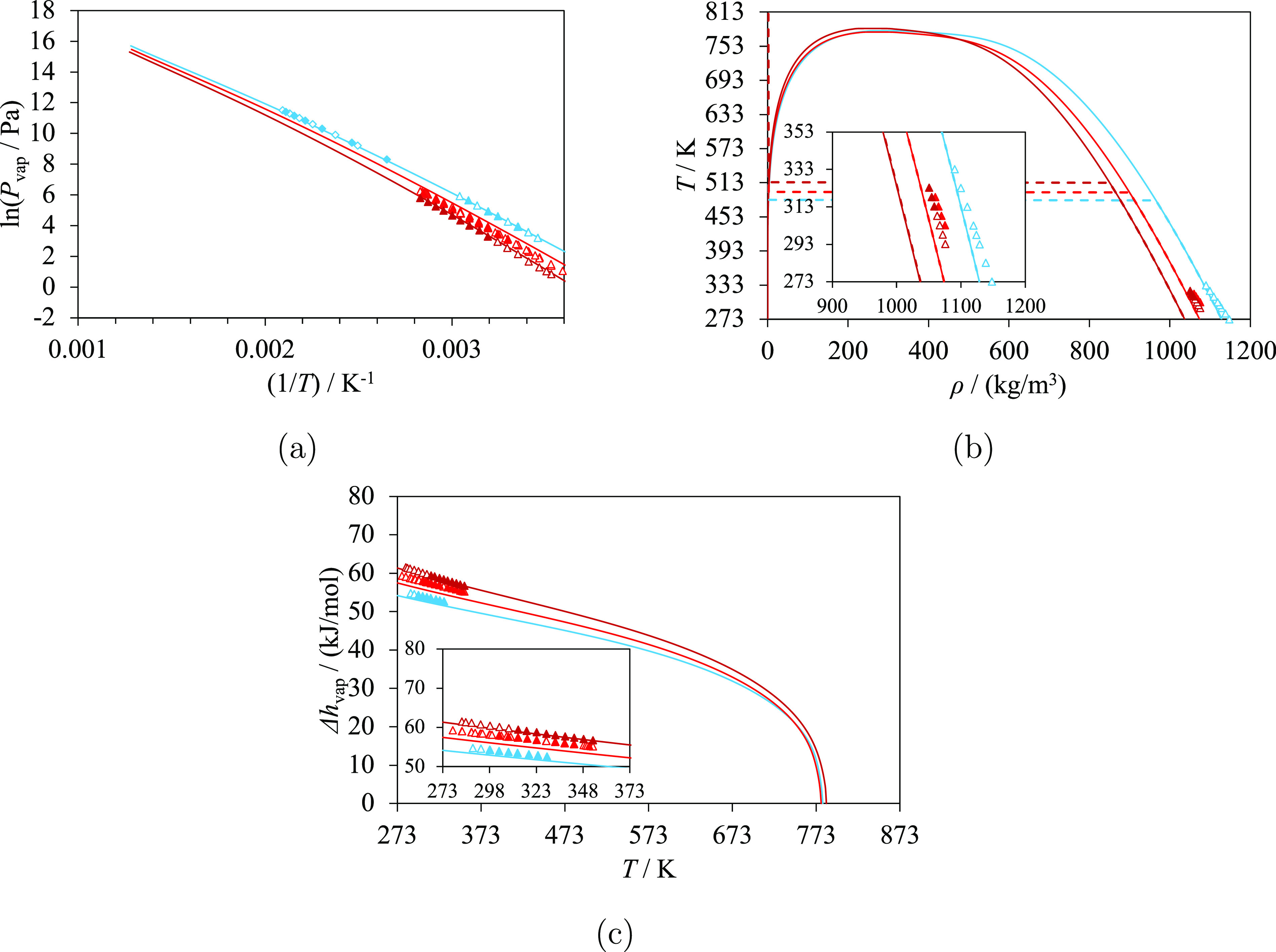
Selected thermodynamic properties of ring lactones: oxolan-2-one (light blue), oxan-2-one (red), and oxepan-2-one (dark red). The curves represent the calculations with SAFT-γ Mie. Experimental data used in the estimation of the group-interaction parameters are represented with filled symbols, and those not used are represented with open symbols. (a) Vapor pressure, with experimental data for oxolan-2-one (triangles^69^ and diamonds^70^), oxan-2-one,^71^ and oxepan-2-one.^72^ (b) Temperature–density diagram, with experimental data for oxolan-2-one,^76^ oxan-2-one,^79^ and oxepan-2-one.^80^ Saturation densities are represented by the continuous curves, and densities at 1 bar are represented by the dashed curves and lines. (c) Vaporization enthalpy, with experimental data for oxolan-2-one,^69^ oxan-2-one,^71^ and oxepan-2-one.^72^ Thermodynamic conditions and the accuracy of the calculations are detailed in Table 7 and in the Zenodo datafile.

We consider experimental data for 5-alkyloxolan-2-ones (from methyl to hexyl chains) and for 6-alkyloxan-2-ones (from methyl to pentyl chains) to characterize the relevant group interactions. The matrix of group interactions used in current and previous work is shown in Table 3, and the like and unlike group parameter values can be found in Tables 4, 5, and 6 with the corresponding references as appropriate. In order to characterize the group interactions, we use pure-component^58,69−82^ and mixture^31,66,83−89^ data of relevant systems. The new group interactions required to model pure lactones and mixtures of lactones and linear alkanes are cCOO–cCOO, cCOO–cCH_2_, cCOO–cCH, cCOO–CH_3_, and cCOO–CH_2_. The results for pure lactones are detailed in the current section; the results for mixtures of lactones and linear alkanes are detailed in the next section. The experimental and calculated vapor pressures (*P*_vap_), single-phase densities (ρ), and vaporization enthalpies (Δ*h*_vap_) for oxolan-2-one, oxan-2-one, and oxepan-2-one are compared in Figure 2. As expected, lower vapor pressures and higher vaporization enthalpies are reported for larger ring sizes. For a given temperature, the vapor pressure and liquid-phase density decrease with increasing ring size of the lactone, while the vaporization enthalpy increases with increasing ring size. Good agreement between the calculations and experiments can be seen, although we note that limited data are available for these molecules.

**Table 3:**
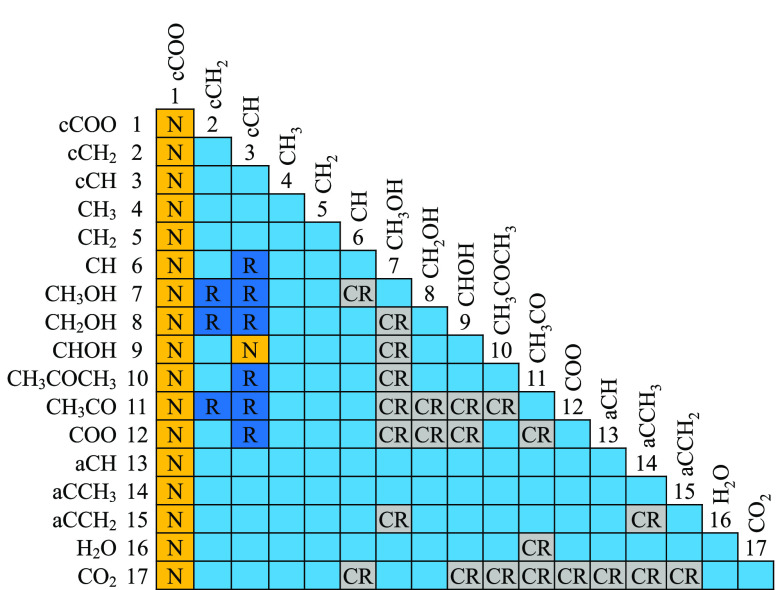
Group Interactions Used to Model Saturated Lactones in a Range of Solvents With the SAFT-γ Mie Approach^a^

a Blue shading denotes that group interaction parameters have been optimized in previous work. The new (N) optimized parameters developed in our current work are denoted by yellow shading. Dark blue shading denotes unlike interaction parameters developed in a separate recent work (R) to be published.^68^ Gray shading denotes that unlike interaction parameters are predicted using combining rules (CR).^8^

**Table 4:** SAFT-γ Mie Group Like Parameters of the Groups Considered in Our Current Work (Excluding Association)^a^

*k*	group	ν_k_^*^	*S*_*k*_	σ_*kk*_/Å	λ_*kk*_^r^	λ_*kk*_^a^	(ε_*kk*_/*k*_B_)/K	*N*_ST,*k*_	*n*_*k*,H_	*n*_*k*_, e_1_	*n*_*k*_, e_2_	ref
1	cCOO	2	0.5787	3.2787	11.855	6.0000	763.67	1	−	2	−	*
2	cCH_2_	1	0.2475	4.7852	20.386	6.0000	477.36	−	−	−	−	(51)
3	cCH	1	0.0961	5.4116	8.0000	6.0000	699.92	−	−	−	−	(41)
4	CH_3_	1	0.5726	4.0772	15.050	6.0000	256.77	−	−	−	−	(8)
5	CH_2_	1	0.2293	4.8801	19.871	6.0000	473.39	−	−	−	−	(8)
6	CH	1	0.0721	5.2950	8.0000	6.0000	95.621	−	−	−	−	(51)
7	CH_3_OH	2	0.8352	3.2462	19.235	6.0000	307.69	2	1	2	−	(51)
8	CH_2_OH	2	0.5854	3.4054	22.699	6.0000	407.22	2	1	2	−	(36)
9	CHOH	2	0.1896	4.5381	18.185	6.0000	599.66	2	1	2	−	(11)
10	CH_3_COCH_3_	3	0.7214	3.5981	17.433	6.0000	286.02	3	1	1	1	(51)
11	CH_3_CO	2	0.5469	3.9112	19.050	6.0000	525.22	1	−	2	−	(39)
12	COO	1	0.6526	3.9939	31.189	6.0000	868.92	1	−	2	−	(8)
13	aCH	1	0.3218	4.0578	14.756	6.0000	371.53	1	−	1	−	(51)
14	aCCH_3_	1	0.3166	5.4874	23.627	6.0000	651.41	1	−	1	−	(37,90)
15	aCCH_2_	1	0.2086	5.2648	8.5433	6.0000	591.56	1	−	1	−	(51)
16	H_2_O	1	1.0000	3.0063	17.020	6.0000	266.68	2	2	2	−	(9,10)
17	CO_2_	2	0.8468	3.0500	26.408	5.0550	207.89	2	−	1	1	(90)

a The asterisk * in the ref column indicates that the cCOO group is characterized in the current work.

**Table 5:** Unlike Group Parameters (Excluding Association) for Use with the SAFT-γ Mie Approach^a^

*k*	*l*	group *k*	group *l*	(ε_*kl*_/*k*_B_)/K	λ_*kl*_^r^	ref
1	1	cCOO	cCOO	763.67	11.855	*
1	2	cCOO	cCH_2_	414.07	11.043	*
1	3	cCOO	cCH	519.28	8.2819	*
1	4	cCOO	CH_3_	411.61	33.394	*
1	5	cCOO	CH_2_	618.67	31.395	*
1	6	cCOO	CH	0.0000	CR	*
1	7	cCOO	CH_3_OH	434.59	CR	*
1	8	cCOO	CH_2_OH	500.14	CR	*
1	9	cCOO	CHOH	373.30	CR	*
1	10	cCOO	CH_3_COCH_3_	482.68	CR	*
1	11	cCOO	CH_3_CO	625.98	CR	*
1	12	cCOO	COO	756.57	9.9399	*
1	13	cCOO	aCH	543.64	17.749	*
1	14	cCOO	aCCH_3_	460.79	9.2834	*
1	15	cCOO	aCCH_2_	464.16	8.0000	*
1	16	cCOO	H_2_O	419.86	CR	*
1	17	cCOO	CO_2_	261.21	8.0000	*
2	2	cCH_2_	cCH_2_	477.36	20.386	(51)
2	3	cCH_2_	cCH	321.71	CR	(41)
2	4	cCH_2_	CH_3_	355.95	CR	(51)
2	5	cCH_2_	CH_2_	469.67	CR	(51)
2	6	cCH_2_	CH	570.45	CR	(51)
2	7	cCH_2_	CH_3_OH	361.42	CR	(68)
2	8	cCH_2_	CH_2_OH	424.64	CR	(68)
2	9	cCH_2_	CHOH	554.50	CR	(11)
2	10	cCH_2_	CH_3_COCH_3_	352.19	CR	(11)
2	11	cCH_2_	CH_3_CO	435.86	CR	(68)
2	12	cCH_2_	COO	498.60	CR	(36)
2	13	cCH_2_	aCH	393.05	15.377	(39)
2	14	cCH_2_	aCCH_3_	540.63	21.082	(39)
2	15	cCH_2_	aCCH_2_	439.78	9.9058	(39)
2	16	cCH_2_	H_2_O	350.99	28.000	(41)
2	17	cCH_2_	CO_2_	269.68	CR	(42)
3	3	cCH	cCH	699.92	8.0000	(41)
3	4	cCH	CH_3_	690.17	CR	(41)
3	5	cCH	CH_2_	522.57	CR	(41)
3	6	cCH	CH	0.0000	CR	(68)
3	7	cCH	CH_3_OH	345.00	CR	(68)
3	8	cCH	CH_2_OH	242.45	CR	(68)
3	9	cCH	CHOH	640.38	CR	*
3	10	cCH	CH_3_COCH_3_	409.28	CR	(68)
3	11	cCH	CH_3_CO	265.24	CR	(68)
3	12	cCH	COO	0.0000	CR	(68)
3	13	cCH	aCH	377.21	CR	(39)
3	14	cCH	aCCH_3_	792.29	CR	(39)
3	15	cCH	aCCH_2_	162.65	CR	(39)
3	16	cCH	H_2_O	377.16	22.265	(41)
3	17	cCH	CO_2_	294.99	CR	(42)
4	4	CH_3_	CH_3_	256.77	15.050	(8)
4	5	CH_3_	CH_2_	350.77	CR	(8)
4	6	CH_3_	CH	387.48	CR	(51)
4	7	CH_3_	CH_3_OH	275.76	15.537	(51)
4	8	CH_3_	CH_2_OH	333.20	CR	(36)
4	9	CH_3_	CHOH	479.38	CR	(11)
4	10	CH_3_	CH_3_COCH_3_	233.48	14.449	(51)
4	11	CH_3_	CH_3_CO	344.57	CR	(39)
4	12	CH_3_	COO	402.75	CR	(8)
4	13	CH_3_	aCH	305.81	CR	(51)
4	14	CH_3_	aCCH_3_	358.58	CR	(36)
4	15	CH_3_	aCCH_2_	396.91	CR	(51)
4	16	CH_3_	H_2_O	358.18	100.00	(36)
4	17	CH_3_	CO_2_	205.70	CR	(90)
5	5	CH_2_	CH_2_	473.39	19.871	(8)
5	6	CH_2_	CH	506.21	CR	(51)
5	7	CH_2_	CH_3_OH	341.41	17.050	(51)
5	8	CH_2_	CH_2_OH	423.17	CR	(36)
5	9	CH_2_	CHOH	517.64	CR	(11)
5	10	CH_2_	CH_3_COCH_3_	299.48	11.594	(51)
5	11	CH_2_	CH_3_CO	431.49	CR	(39)
5	12	CH_2_	COO	498.86	CR	(8)
5	13	CH_2_	aCH	415.64	CR	(51)
5	14	CH_2_	aCCH_3_	569.18	CR	(36)
5	15	CH_2_	aCCH_2_	454.16	CR	(51)
5	16	CH_2_	H_2_O	423.63	100.00	(36)
5	17	CH_2_	CO_2_	276.45	CR	(90)
6	6	CH	CH	95.621	8.0000	(51)
6	7	CH	CH_3_OH	CR	CR	−
6	8	CH	CH_2_OH	329.22	CR	(37)
6	9	CH	CHOH	0.0000	CR	(11)
6	10	CH	CH_3_COCH_3_	637.29	CR	(51)
6	11	CH	CH_3_CO	321.91	CR	(39)
6	12	CH	COO	353.65	CR	(37)
6	13	CH	aCH	441.43	CR	(51)
6	14	CH	aCCH_3_	769.36	8.0000	(39)
6	15	CH	aCCH_2_	65.410	CR	(51)
6	16	CH	H_2_O	275.75	CR	(37)
6	17	CH	CO_2_	CR	CR	−
7	7	CH_3_OH	CH_3_OH	307.69	19.235	(51)
7	8	CH_3_OH	CH_2_OH	CR	CR	−
7	9	CH_3_OH	CHOH	CR	CR	−
7	10	CH_3_OH	CH_3_COCH_3_	CR	CR	−
7	11	CH_3_OH	CH_3_CO	CR	CR	−
7	12	CH_3_OH	COO	CR	CR	−
7	13	CH_3_OH	aCH	330.19	CR	(39)
7	14	CH_3_OH	aCCH_3_	405.45	CR	(39)
7	15	CH_3_OH	aCCH_2_	CR	CR	−
7	16	CH_3_OH	H_2_O	278.45	CR	(51)
7	17	CH_3_OH	CO_2_	157.83	8.3462	(90)
8	8	CH_2_OH	CH_2_OH	407.22	22.699	(36)
8	9	CH_2_OH	CHOH	389.23	CR	(11)
8	10	CH_2_OH	CH_3_COCH_3_	338.47	CR	(11)
8	11	CH_2_OH	CH_3_CO	CR	CR	−
8	12	CH_2_OH	COO	CR	CR	−
8	13	CH_2_OH	aCH	386.05	CR	(37)
8	14	CH_2_OH	aCCH_3_	486.62	CR	(37)
8	15	CH_2_OH	aCCH_2_	434.37	CR	(37)
8	16	CH_2_OH	H_2_O	353.37	CR	(36)
8	17	CH_2_OH	CO_2_	312.30	CR	(42)
9	9	CHOH	CHOH	599.66	18.185	(11)
9	10	CHOH	CH_3_COCH_3_	340.81	CR	(11)
9	11	CHOH	CH_3_CO	CR	CR	−
9	12	CHOH	COO	CR	CR	−
9	13	CHOH	aCH	512.16	CR	(11)
9	14	CHOH	aCCH_3_	762.86	CR	(11)
9	15	CHOH	aCCH_2_	357.91	CR	(11)
9	16	CHOH	H_2_O	479.16	CR	(11)
9	17	CHOH	CO_2_	CR	CR	−
10	10	CH_3_COCH_3_	CH_3_COCH_3_	286.02	17.433	(51)
10	11	CH_3_COCH_3_	CH_3_CO	CR	CR	−
10	12	CH_3_COCH_3_	COO	547.44	CR	(11)
10	13	CH_3_COCH_3_	aCH	333.11	CR	(51)
10	14	CH_3_COCH_3_	aCCH_3_	479.55	35.957	(39)
10	15	CH_3_COCH_3_	aCCH_2_	394.83	CR	(51)
10	16	CH_3_COCH_3_	H_2_O	287.26	CR	(51)
10	17	CH_3_COCH_3_	CO_2_	CR	CR	−
11	11	CH_3_CO	CH_3_CO	525.22	19.050	(39)
11	12	CH_3_CO	COO	CR	CR	
11	13	CH_3_CO	aCH	426.72	18.030	(39)
11	14	CH_3_CO	aCCH_3_	552.62	36.429	(39)
11	15	CH_3_CO	aCCH_2_	663.71	30.712	(39)
11	16	CH_3_CO	H_2_O	CR	CR	−
11	17	CH_3_CO	CO_2_	CR	CR	−
12	12	COO	COO	868.92	31.189	(8)
12	13	COO	aCH	534.18	22.088	(39)
12	14	COO	aCCH_3_	595.48	15.874	(39)
12	15	COO	aCCH_2_	265.62	9.3393	(39)
12	16	COO	H_2_O	396.81	15.140	(91)
12	17	COO	CO_2_	CR	CR	−
13	13	aCH	aCH	371.53	14.756	(51)
13	14	aCH	aCCH_3_	471.23	CR	(90)
13	15	aCH	aCCH_2_	416.69	CR	(51)
13	16	aCH	H_2_O	357.78	38.640	(51)
13	17	aCH	CO_2_	CR	CR	−
14	14	aCCH_3_	aCCH_3_	651.41	23.627	(37,90)
14	15	aCCH_3_	aCCH_2_	CR	CR	−
14	16	aCCH_3_	H_2_O	360.70	CR	(37)
14	17	aCCH_3_	CO_2_	CR	CR	−
15	15	aCCH_2_	aCCH_2_	591.56	8.5433	(51)
15	16	aCCH_2_	H_2_O	329.03	CR	(37)
15	17	aCCH_2_	CO_2_	CR	CR	−
16	16	H_2_O	H_2_O	266.68	17.020	(9,10)
16	17	H_2_O	CO_2_	226.38	CR	(90)
17	17	CO_2_	CO_2_	207.89	26.408	(90)

a The asterisk * indicates that the parameters are characterized in the current work.

**Table 6:** Group Association Parameters for Use with the SAFT-γ Mie Approach^a^

*k*	*l*	group *k*	site *a* of group *k*	group *l*	site *b* of group *l*	(ε_*kl*,*ab*_^HB^/*k*_B_)/K	*K*_*kl*,*ab*_^HB^/Å^3^	ref
1	7	cCOO	e_1_	CH_3_OH	H	1267.6	1095.9	*
1	8	cCOO	e_1_	CH_2_OH	H	1496.8	626.15	*
1	9	cCOO	e_1_	CHOH	H	1674.2	635.00	*
1	16	cCOO	e_1_	H_2_O	H	1385.6	576.40	*
7	7	CH_3_OH	H	CH_3_OH	e_1_	2062.1	106.57	(51)
7	16	CH_3_OH	e_1_	H_2_O	H	1993.5	104.11	(51)
7	16	CH_3_OH	H	H_2_O	e_1_	1993.5	104.11	(51)
8	8	CH_2_OH	H	CH_2_OH	e_1_	2097.9	62.309	(36)
8	9	CH_2_OH	H	CHOH	e_1_	2500.0	10.444	(11)
8	9	CH_2_OH	e_1_	CHOH	H	1464.1	591.55	(11)
8	10	CH_2_OH	e_1_	CH_3_COCH_3_	H	686.93	585.99	(11)
8	10	CH_2_OH	H	CH_3_COCH_3_	e_1_	1844.8	991.95	(11)
8	16	CH_2_OH	e_1_	H_2_O	H	2153.2	147.40	(36)
8	16	CH_2_OH	H	H_2_O	e_1_	621.68	425.00	(36)
9	9	CHOH	H	CHOH	e_1_	2480.6	8.4740	(11)
9	10	CHOH	e_1_	CH_3_COCH_3_	H	1186.9	731.08	(11)
9	10	CHOH	H	CH_3_COCH_3_	e_1_	1323.1	635.37	(11)
9	16	CHOH	e_1_	H_2_O	H	2140.9	19.478	(11)
9	16	CHOH	H	H_2_O	e_1_	2289.1	63.813	(11)
10	10	CH_3_COCH_3_	H	CH_3_COCH_3_	e_1_	980.20	2865.2	(51)
10	16	CH_3_COCH_3_	H	H_2_O	e_1_	1386.8	188.83	(51)
10	16	CH_3_COCH_3_	e_1_	H_2_O	H	1588.7	772.77	(51)
10	16	CH_3_COCH_3_	e_2_	H_2_O	H	417.24	1304.3	(51)
12	16	COO	e_1_	H_2_O	H	1245.8	454.98	(91)
13	16	aCH	e_1_	H_2_O	H	563.56	339.61	(51)
14	16	aCCH_3_	e_1_	H_2_O	H	563.56	339.61	(37)
15	16	aCCH_2_	e_1_	H_2_O	H	563.56	339.61	(37)
16	16	H_2_O	H	H_2_O	e_1_	1985.4	101.69	(9,10)
16	17	H_2_O	e_1_	CO_2_	e_1_	1398.1	91.419	(90)

a The asterisk * indicates that the parameters are characterized in the current work.

**Figure 3:**
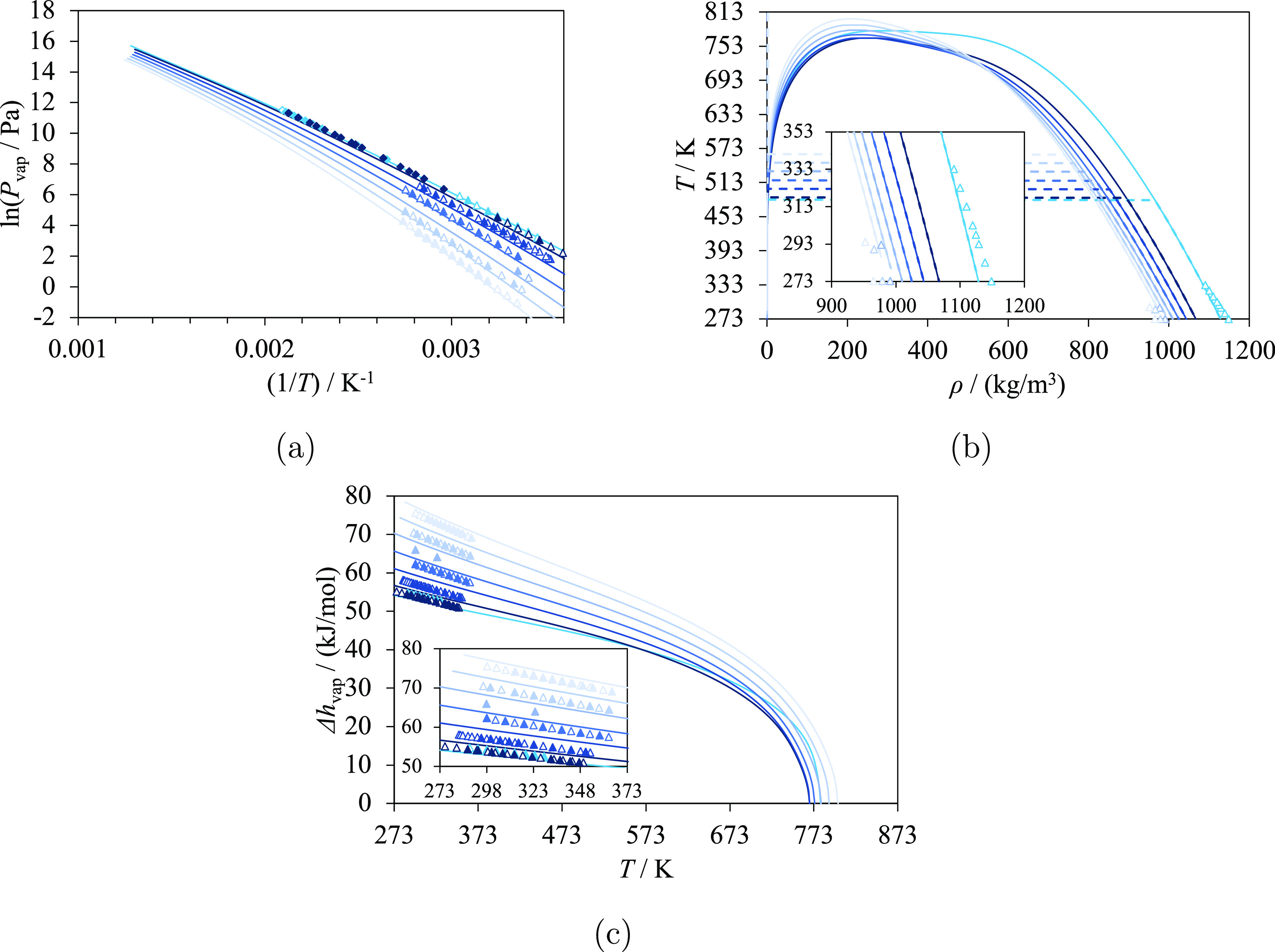
Selected thermodynamic properties of oxolan-2-one (light blue) and alkyloxolan-2-ones: 5-methyloxolan-2-one, 5-ethyloxolan-2-one, 5-propyloxolan-2-one, 5-butyloxolan-2-one, 5-pentyloxolan-2-one, and 5-hexyloxolan-2-one (from dark blue to pale blue). The curves represent the calculations with SAFT-γ Mie. Experimental data used in the estimation of group-interaction parameters are represented with filled symbols, and those not used are represented with open symbols. (a) Vapor pressure, with experimental data of oxolan-2-one (triangles^69^ and diamonds^70^), 5-methyloxolan-2-one (triangles^58^ and diamonds^73^), 5-ethyloxolan-2-one,^74^ 5-propyloxolan-2-one,^74^ 5-butyloxolan-2-one,^75^ 5-pentyloxolan-2-one,^74^ and 5-hexyloxolan-2-one.^74^ (b) Temperature–density diagram, with experimental data of oxolan-2-one,^76^ 5-methyloxolan-2-one,^73^ 5-butyloxolan-2-one,^81^ 5-pentyloxolan-2-one,^81^ and 5-hexyloxolan-2-one.^81^ Saturation densities are represented by the continuous curves, and densities at 1 bar are represented by the dashed curves and lines. (c) Vaporization enthalpy, with experimental data of oxolan-2-one,^69^ 5-methyloxolan-2-one,^74^ 5-ethyloxolan-2-one,^74^ 5-propyloxolan-2-one,^74^ 5-butyloxolan-2-one,^75^ 5-pentyloxolan-2-one,^74^ and 5-hexyloxolan-2-one.^74^ Thermodynamic conditions and the accuracy of the calculations are detailed in Table 7 and in the Zenodo datafile.

**Figure 4:**
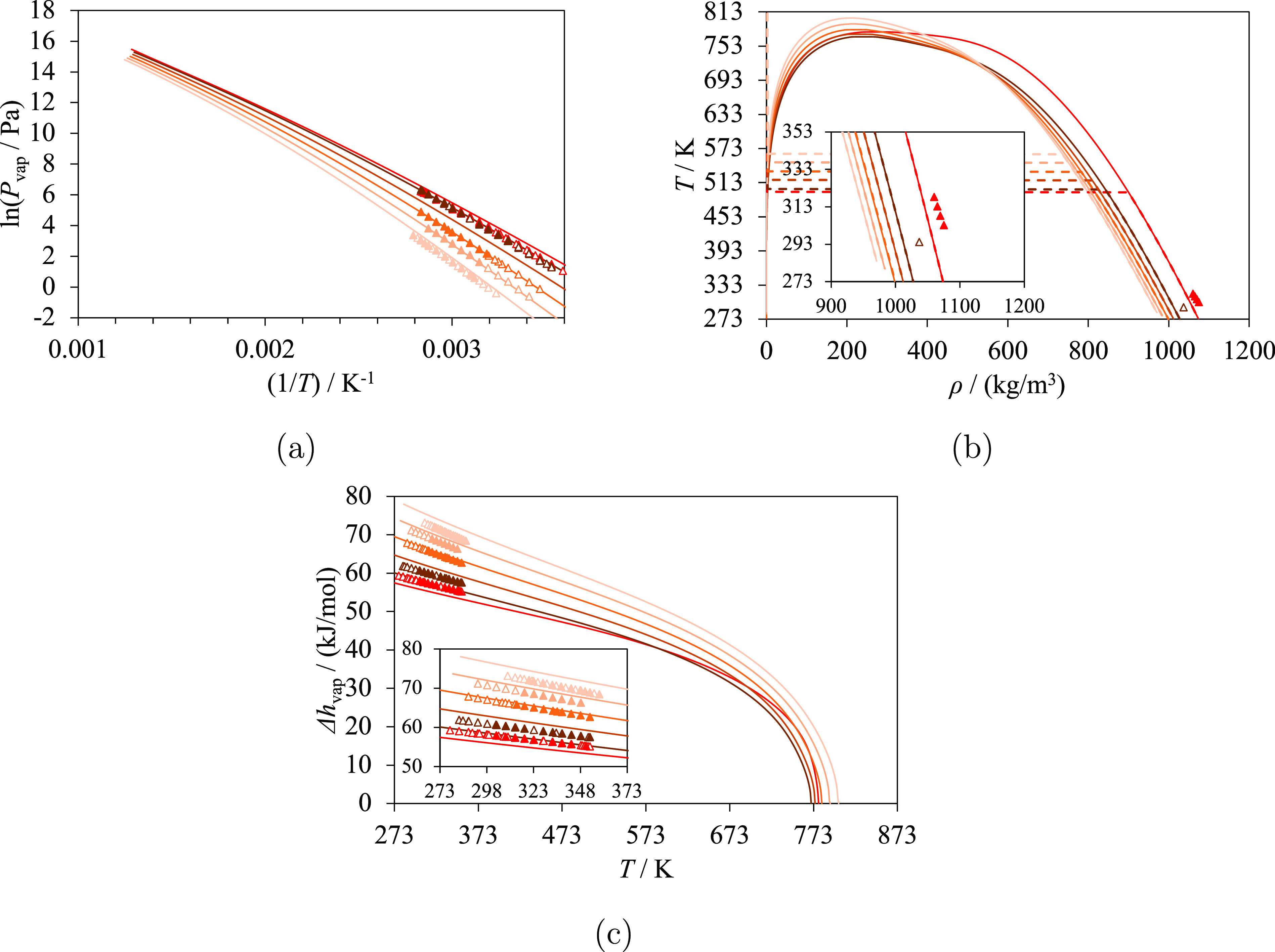
Selected thermodynamic properties of oxan-2-one (red) and alkyloxan-2-ones: 6-methyloxan-2-one, 6-ethyloxan-2-one, 6-propyloxan-2-one, 6-butyloxan-2-one, and 6-pentyloxan-2-one (from dark orange to pale orange). The curves represent the calculations with SAFT-γ Mie. Experimental data used in the estimation of group-interaction parameters are represented with filled symbols, and those not used are represented with open symbols. (a) Vapor pressure, with experimental data of oxan-2-one,^71^ 6-methyloxan-2-one,^71^ 6-propyloxan-2-one,^71^ 6-butyloxan-2-one,^71^ and 6-pentyloxan-2-one.^71^ (b) Temperature–density diagram, with experimental data of oxan-2-one,^71^ and 6-methyloxan-2-one.^82^ Saturation densities are represented by the solid curves, and densities at 1 bar are represented by the dashed curves and line. (c) Vaporization enthalpy, with experimental data of oxan-2-one,^71^ 6-methyloxan-2-one,^71^ 6-propyloxan-2-one,^71^ 6-butyloxan-2-one,^71^ and 6-pentyloxan-2-one.^71^ Thermodynamic conditions and accuracy are detailed in Table 7 and in the Zenodo datafile.

The family of 5-alkyloxolan-2-ones corresponds to lactones with five atoms in the lactone ring and an alkyl side chain (in position 5) next to the oxygen of the ring (position 1). Similarly, the family of 6-alkyloxan-2-ones corresponds to lactones with six atoms in the lactone ring and an alkyl side chain (in position 6). The calculated vapor pressures, single-phase densities, and vaporization enthalpies are compared with the corresponding experimental data for pure 5-alkyloxolan-2-ones and 6-alkyloxan-2-ones with several side-chain lengths in Figure 3 and Figure 4, respectively. The vapor pressure and liquid-phase density decrease with increasing length of the side chain, while the vaporization enthalpy increases with increasing length of the side chain. Very good quantitative agreement can be seen for alkyllactones although the comparison with experimental data is possible only for a small temperature range, far from the critical temperature.

We report %AAD and AAD in order to assess the performance of our model. It is especially relevant to consider both measures, given the limited data available for comparison and the low-temperature and pressure nature of the data. The accuracy of the results for pure lactones is summarized in Table 7, where %AADs and AADs can be found for each system and property. The %AADs for the vapor pressure of oxolan-2-one and oxepan-2-one are both smaller than 10% (5.063%, and 7.853%, respectively). The AAD obtained for the vapor pressure of oxolan-2-one (1796 Pa) is, however, much larger than the AAD obtained for oxepan-2-one (5.722 Pa) because of the difference in the temperature ranges (289–478 and 283–353 K, respectively). The largest %AAD shown in Table 7 is for the vapor pressure of oxan-2-one (44.58%) but corresponds to a small AAD (46.23 Pa). The largest AAD is obtained for the vapor pressure of 5-methyloxolan-2-one (1929 Pa) because of the large temperature range (308–470 K). All %AADs for the liquid-phase densities and the vaporization enthalpy of the pure lactones considered are smaller than 5%. In absolute terms, this corresponds to a maximum AAD in liquid-phase densities for oxepan-2-one (51.40 kg/m^3^) and a maximum AAD in vaporization enthalpy for 6-pentyloxan-2-one (2441 J/mol).

**Table 7:** Overview of the Accuracy of SAFT-γ Mie in the Calculation of Vapor Pressures *P*_vap_(*T*), Densities ρ(*T*,*P*), and Vaporization Enthalpies Δ*h*_vap_(*T*) for Pure Lactones, Where *N*_*s*,*p*_^D^ Is the Number of Experimental Data Points Used in the Parameter Estimation, and *N*_*s*,*p*_^D,total^ Is the Number of Experimental Data Used to Calculate %AAD_*s*_*p* and AAD_*s*_*p* for System *s* and Property *p*

compound *s*	*T*/K range	*N*_*s*,*p*_^D^	*N*_*s*,*p*_^D,total^	%AAD_*s*_*P*_vap_(*T*)	AAD*_s_P*_vap_ (*T*)/Pa	figure	ref
oxolan-2-one	289–478	11	26	5.063	1796	2(a,b), 3(a,b)	(69,70)
oxan-2-one	278–353	9	51	44.58	46.23	2(a,b)	(71)
oxepan-2-one	283–353	9	16	7.853	5.722	2(a,b)	(72)
5-methyloxolan-2-one	308–470	9	33	25.17	1929	3(a,b)	(58,73)
5-ethyloxolan-2-one	283–353	9	24	32.58	41.39	3(a,b)	(74)
5-propyloxolan-2-one	298–363	7	14	22.26	31.59	3(a,b)	(74)
5-butyloxolan-2-one	293–298	1	2	28.76	0.6564	3(a,b)	(75)
5-pentyloxolan-2-one	296–363	7	14	21.01	6.107	3(a,b)	(74)
5-hexyloxolan-2-one	298–365	10	23	17.58	2.262	3(a,b)	(74)
6-methyloxan-2-one	283–353	10	29	10.09	4.150	4(a,b)	(71)
6-propyloxan-2-one	288–353	10	18	1.663	0.3405	4(a,b)	(71)
6-butyloxan-2-one	293–348	7	12	8.045	0.6939	4(a,b)	(71)
6-pentyloxan-2-one	309–358	10	23	30.63	3.206	4(a,b)	(71)

compound *s*	*T*/K range	*P*/MPa range	*N*_*s*,*p*_^D^	*N*_*s*,*p*_^D,total^	%AAD_*s*_ ρ(*T*, *P*)	AAD_*s*_ ρ(*T*,*P*)/(kg/m^3^)	figure	ref
oxolan-2-one	253–473	0.101–35.0	13	363	1.021	10.90	2(c), 3(c)	(76,77)
oxan-2-one	293–318	0.101–10.0	4	10	3.416	37.62	2(c)	(78),^79^
oxepan-2-one	293–323	0.100	3	7	4.834	51.40	2(c)	(80)
5-methyloxolan-2-one	293–453	0.186–0.983	14	72	1.257	11.92	3(c)	(73)
5-butyloxolan-2-one	273–293	0.101	0	2	1.838	18.09	3(c)	(81)
5-pentyloxolan-2-one	273–290	0.101	0	2	1.870	18.18	3(c)	(81)
5-hexyloxolan-2-one	273–294	0.101	0	2	2.109	20.22	3(c)	(81)
6-methyloxan-2-one	294	0.101	0	1	2.399	24.87	4(c)	(82)

compound *s*	*T*/K range	*Ν*_*s*,*p*_^D^	*N*_*s*,*p*_^D,total^	%AAD_*s*_ Δ*h*_vap_(*T*)	AAD_*s*_ Δ*h*_vap_ (*T*)/(J/mol)	figure	ref
oxolan-2-one	289–329	10	14	2.462	1329	2(d), 3(d)	(69)
oxan-2-one	278–353	9	51	3.659	2095	2(d)	(71)
oxepan-2-one	283–353	9	16	0.7581	454.7	2(d)	(72)
5-methyloxolan-2-one	276–350	10	22	2.751	1453	3(d)	(74)
5-ethyloxolan-2-one	283–353	9	24	4.048	2273	3(d)	(74)
5-propyloxolan-2-one	298–363	7	14	2.349	1407	3(d)	(74)
5-butyloxolan-2-one	298–324	2	2	3.159	2054	3(d)	(75)
5-pentyloxolan-2-one	296–363	7	14	3.400	2291	3(d)	(74)
5-hexyloxolan-2-one	298–365	10	23	2.141	1546	3(d)	(74)
6-methyloxan-2-one	283–353	10	29	3.988	2388	4(d)	(71)
6-propyloxan-2-one	288–353	10	18	0.6015	392.0	4(d)	(71)
6-butyloxan-2-one	293–348	7	12	1.865	1281	4(d)	(71)
6-pentyloxan-2-one	309–358	10	23	3.458	2441	4(d)	(71)

#### 3.1.2 Binary Mixtures of Lactones + Linear/Cyclic Alkanes

We now consider binary mixtures of lactones with cyclohexane or *n*-alkanes; the groups involved in these mixtures are the same as the groups involved in pure lactones. Specifically, we consider mixtures containing linear *n*-alkanes from *n*-hexane to *n*-octane and from *n*-undecane to *n*-nonadecane. Cyclohexane is modeled using six cCH_2_ groups; linear alkanes comprise CH_2_ and CH_3_ groups. As mentioned in the previous section, the experimental data related to these mixtures (summarized in Table 8) are used together with the experimental data for pure lactones (cf. Table 7) to estimate the parameters of the cCOO group and the unlike interactions (specifically for cCOO–CH_2_ and cCOO–CH_3_).

**Table 8:** Overview of the Accuracy of SAFT-γ Mie in the Calculation of the Azeotrope Composition *x*_oxolan-2-one_^az^ and Temperature *T*^az^, Liquid–Liquid Equilibrium Compositions *x*_1_^LLE^, Solubilities *x*_1_^sat^, and Excess Molar Enthalpies Δ*h*_mix_ for Binary Mixtures of Lactones and Hydrocarbons, Where *N*_*s*,*p*_^D^ Is the Number of Experimental Data Used in the Parameter Estimation, and *N*_*s*,*p*_^D,total^ Is the Number of Experimental Data Used to Calculate %AAD_*s*_*p* and AAD_*s*_*p* for System *s* and Property *p*

system *s* (1 + 2)	*T*/K range	*P*/MPa range	*x*_1_ range	*N*_*s*,*p*_^D^	*N*_*s*,*p*_^total^	%AAD_*s*_*x*_oxolan-2-one_^az^	AAD_*s*_*x*_oxolan-2-one_^az^	figure	ref
oxolan-2-one + *n*-octane	−	0.101	−	0	1	10.34	0.007960	5(e), 6(a)	(83)
oxolan-2-one + *n*-undecane	−	0.00267	−	0	1	1.883	0.008285	6(a)	(83)
oxolan-2-one + *n*-dodecane	−	0.00267	−	0	1	1.241	0.008252	6(a)	(83)
oxolan-2-one + *n*-tridecane	−	0.00267	−	0	1	1.635	0.01329	6(a)	(83)
oxolan-2-one + *n*-tetradecane	−	0.00267	−	0	1	0.9825	0.008941	6(a)	(83)
oxolan-2-one + *n*-pentadecane	−	0.00267	−	0	1	1.470	0.01417	6(a)	(83)
oxolan-2-one + *n*-hexadecane	−	0.00267	−	0	1	1.075	0.01062	6(a)	(83)
oxolan-2-one + *n*-heptadecane	−	0.00267	−	0	1	0.3060	0.003036	6(a)	(83)
oxolan-2-one + *n*-octadecane	−	0.00267	−	0	1	0.003978	0.00003958	6(a)	(83)
oxolan-2-one + *n*-nonadecane	−	0.00267	−	0	1	0.03086	0.0003080	6(a)	(83)

system *s* (1 + 2)	*T*/K range	*P*/MPa range	*x*_1_ range	*N*_*s*,*p*_^D^	*N*_*s*,*p*_^total^	%AAD_*s*_*T*^az^	AAD_*s*_*T*^az^/K	figure	ref
oxolan-2-one + *n*-octane	−	0.101	−	0	1	0.01256	0.05	6(b)	(83)
oxolan-2-one + *n*-undecane	−	0.00267	−	0	1	1.106	3.85	6(b)	(83)
oxolan-2-one + *n*-tridecane	−	0.00267	−	0	1	0.7870	2.85	6(b)	(83)
oxolan-2-one + *n*-pentadecane	−	0.00267	−	0	1	1.332	4.85	6(b)	(83)
oxolan-2-one + *n*-heptadecane	−	0.00267	−	0	1	1.051	3.85	6(b)	(83)
oxolan-2-one + *n*-nonadecane	−	0.00267	−	0	1	0.7762	2.85	6(b)	(83)

system *s* (1 + 2)	*T*/K range	*P*/MPa range	*x*_1_ range	*N*_*s*,*p*_^D^	*N*_*s*,*p*_^total^	%AAD_*s*_*x*_1_^LLE^	AAD_*s*_*x*_1_^LLE^	figure	ref
oxolan-2-one + cyclohexane	298	0.101	−	2	2	165.3	0.04311	5(a)	(84)
oxepan-2-one + cyclohexane	293–303	0.101	−	2	2	178.3	0.1261	5(b)	(85)
5-methyloxolan-2-one + cyclohexane	288–330	0.101	−	17	17	57.78	0.2114	−	(31)
oxolan-2-one + *n*-hexane	293–298	0.101	−	3	3	44.26	0.02430	5(c)	(84,86)
oxepan-2-one + *n*-hexane	293–323	0.101	−	4	4	15.25	0.005918	5(d)	(85)
oxolan-2-one + *n*-heptane	298	0.101	−	2	2	53.51	0.01953	−	(84)
oxepan-2-one + *n*-heptane	293–323	0.101	−	4	4	23.57	0.02007	−	(85)
5-methyloxolan-2-one + *n*-heptane	288–364	0.101	−	22	22	66.47	0.1179	−	(31)
5-methyloxolan-2-one + *n*-decane	288–358	0.101	−	0	18	135.2	0.07116	−	(31)
5-methyloxolan-2-one + *n*-dodecane	283–358	0.101	−	0	19	163.3	0.06151	−	(31)
oxolan-2-one + *n*-tridecane	293–368	0.101	−	0	10	120.4	0.02407	−	(83)
5-methyloxolan-2-one + *n*-tridecane	283–333	0.101	−	0	20	180.6	0.08207	−	(87)

system *s* (1 + 2)	*T*/K range	*P*/MPa range	*x*_1_ range	*N*_*s*,*p*_^D^	*N*_*s*,*p*_^total^	%AAD_*s*_*x*_1_^sat^	AAD_*s*_*x*_1_^sat^	figure	ref
oxepan-2-one in cyclohexane	267–272	0.101	−	0	4	29.60	0.2510	5(b)	(66)
cyclohexane in oxepan-2-one	270–279	0.101	−	0	7	256.6	0.5212	5(b)	(66)

system *s* (1+2)	*T*/K range	*P*/MPa range	*x*_1_ range	*N*_*s*,*p*_^D^	*N*_*s*,*p*_^total^	%AAD_*s*_ Δ*h*_mix_	AAD_*s*_ Δ*h*_mix_/(J/mol)	figure	ref
oxolan-2-one + cyclohexane	298	0.101	0.982–0.995	6	6	64.96	68.13	−	(88)
oxolan-2-one + *n*-hexane	298	0.101	0.0048–0.014	8	8	6.082	6.705	−	(88)
oxolan-2-one + *n*-heptane	298–318	0.101	0.003–0.9968	11	43	32.59	42.10	−	(89)

**Figure 5:**
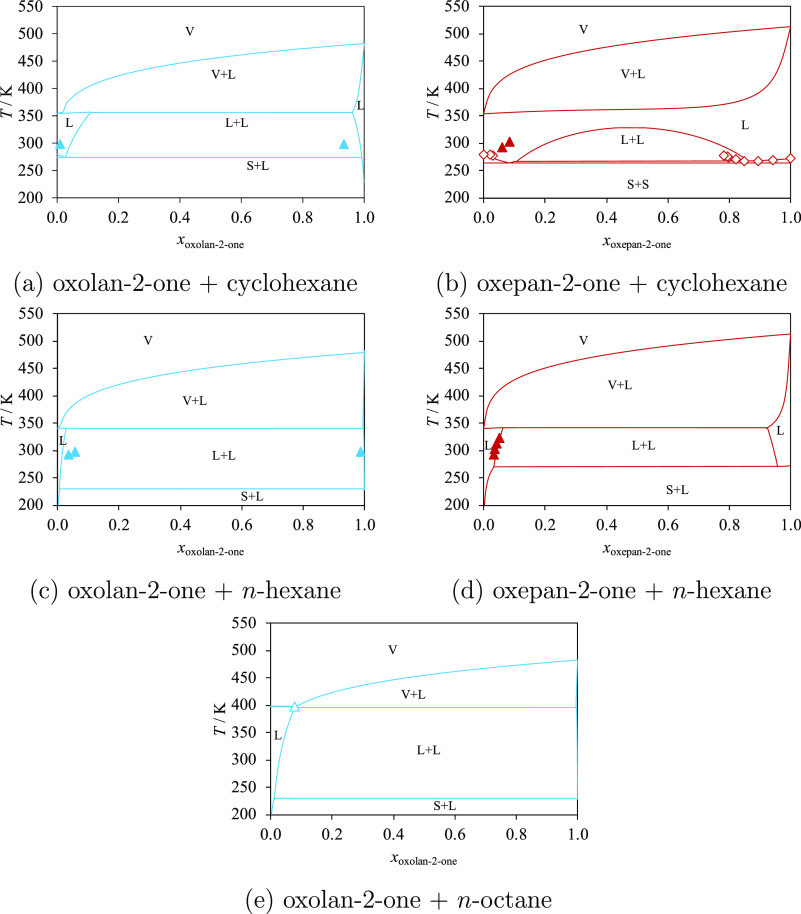
Isobaric phase diagrams of ring lactones + hydrocarbons: oxolan-2-one (light blue) and oxepan-2-one (dark red) at atmospheric pressure. The curves represent the calculations with SAFT-γ Mie. The letters V, L and S indicate vapor, liquid, and solid phases, respectively. Experimental data used in the estimation of group-interaction parameters are represented with filled symbols, and those not used are represented with open symbols. (a) Oxolan-2-one + cyclohexane, with experimental data for the LLE.^84^ (b) Oxepan-2-one + cyclohexane, with experimental data for the LLE^85^ and SLE.^66^ (c) Oxolan-2-one + *n*-hexane, with experimental data for the LLE.^84,86^ (d) Oxepan-2-one + *n*-hexane, with experimental data for LLE.^85^ (e) Oxolan-2-one + *n*-octane, with experimental data for the azeotrope.^83^ Thermodynamic conditions and the accuracy of the calculations are detailed in Table 8 and in the Zenodo datafile.

Isobaric temperature–composition phase diagrams for a number of mixtures of lactones (oxolan-2-one or oxepan-2-one) and hydrocarbons (cyclohexane, *n*-hexane, or *n*-octane) are shown in Figure 5, in which SAFT-γ Mie calculations and the limited experimental data available can be compared. Specifically, the experimental LLE data for mixtures of oxolan-2-one and cyclohexane,^84^ oxolan-2-one and *n*-hexane,^84,86^ and oxepan-2-one and *n*-hexane^85^ can be seen in the figures. Good overall agreement is observed, as indicated by the low AADs summarized in Table 8. The small values of the LLE mole fractions cause some large %AADs, for example, for oxolan-2-one + cyclohexane (165.3%), despite a small corresponding AAD (0.04311). Experimental data for LLE^85^ and solid–liquid–liquid equilibrium^66^ (SLLE) are shown for the mixture of oxepan-2-one and cyclohexane in Figure 5b. The model allows for the correct prediction of the existence and the extent of the LLE region for this system but fails in predicting the composition of the eutectic point (*x*_oxepan-2-one_^SAFT^ = 0.084 and *x*_oxepan-2-one_^exp^ = 0.847), in part as a consequence of the small difference in the SLLE temperature and the eutectic temperature. The temperature of the eutectic point predicted by SAFT-γ Mie with the estimated parameters is *T*_eutectic_^SAFT^ = 264 K, while the experimental value^66^ is *T*_eutectic_^exp^ = 267 K.

**Figure 6:**
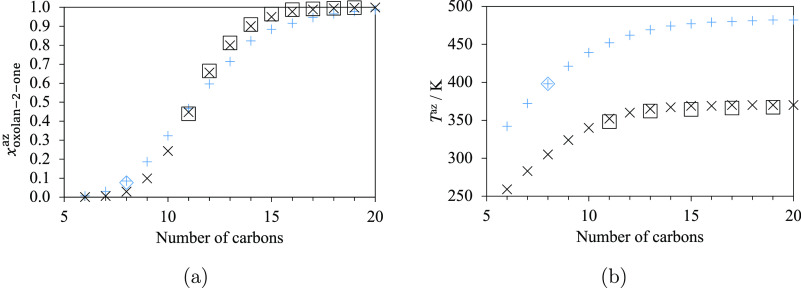
(a) Azeotrope compositions given as the mole fraction of oxolan-2-one (*x*_oxolan-2-one_^az^) and (b) azeotrope temperatures (*T*^az^) for mixtures of oxolan-2-one + linear alkanes. The number of carbons corresponds to the length of the alkane (six for *n*-hexane, seven for *n*-heptane, etc.). Black “×” symbols and blue “+” symbols represent the SAFT-γ Mie calculations. Black squares and blue diamonds represent the experimental points.^83^ Black points and blue points correspond to results at 2.6664 and 101.3 kPa, respectively. Thermodynamic conditions and the accuracy of the calculations are detailed in Table 8 and in the Zenodo datafile.

Several azeotrope data are available for mixtures of oxolan-2-one and linear alkanes.^83^ This allows us to study the influence of the alkane chain length on the azeotrope composition and temperature at 2666.4 Pa. Related experimental data are available for systems containing alkanes from *n*-undecane (C_11_H_24_) to *n*-nonadecane (C_19_H_40_). SAFT-γ Mie calculations are carried out for *n*-hexane (C_6_H_14_) to *n*-eicosane (C_20_H_42_). The azeotrope composition *x*_oxolan-2-one_^az^ increases with an increase in the alkane length (Figure 6a) and is found to be greater than 0.5 for oxolan-2-one + *n*-dodecane (C_12_H_26_) and longer alkanes, both from the experimental data and the predictions. The azeotrope temperature also increases with an increase in the alkane length at a fixed pressure (Figure 6b). The agreement between the experimental data and SAFT-γ Mie predictions is excellent both for the azeotrope compositions and temperatures, with the corresponding %AADs smaller than 2% for all the mixtures at 2.6664 kPa (cf. Table 8). For 101.3 kPa, only one azeotrope temperature has been reported^83^ for the VLE of oxolan-2-one + *n*-octane (Figures 5e and 6a,b), and the SAFT-γ Mie predictions are found to be in excellent agreement with these data. It also can be seen in Figure 6 that the azeotrope temperature of a given mixture increases significantly with an increase in the pressure, while the compositions remain quite similar. Other %AADs and ADDs associated with the mixtures of lactones and hydrocarbons are summarized in Table 8.

### 3.2 Binary Mixtures of Lactones + Alcohols

#### 3.2.1 Lactone + Primary Alcohol Mixtures: cCOO–CH_3_OH and cCOO–CH_2_OH Interactions

Mixtures of lactones and primary alcohols, from methanol to decan-1-ol, are studied in this section. Methanol is represented by a single SAFT-γ Mie molecular group,^51^ denoted as CH_3_OH. Longer primary alcohols (ethanol, propan-1-ol, butan-1-ol, etc.) are modeled with a CH_3_ group, a number of CH_2_ groups appropriate for the length of the alkanol, and a CH_2_OH^36^ group (cf. Table 2). The CH_3_OH and CH_2_OH groups include one site of type H, and two e sites (labeled e_1_) correspond to the lone pairs of the oxygen atom. In mixtures with lactones, an association interaction between the H site of the hydroxyl groups and the e_1_ site of the cCOO group is accounted for. The related parameters, characterized here, are , , , and ; in addition, the unlike dispersion energy parameters  and  are also determined (cf. Tables 5 and 6).

**Figure 7:**
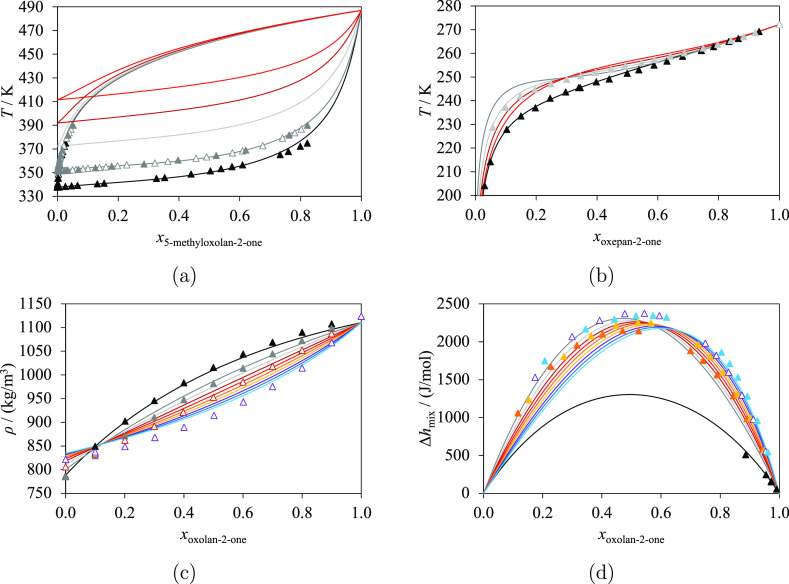
Selected isobaric thermodynamic properties at atmospheric pressure for lactones + primary alcohols: methanol (black), ethanol (dark gray), propan-1-ol (light gray), butan-1-ol (dark red), pentan-1-ol (red), hexan-1-ol (orange), heptan-1-ol (yellow), octan-1-ol (purple), nonan-1-ol (dark blue), and decan-1-ol (light blue). The curves represent the calculations with SAFT-γ Mie. Experimental data used in the estimation of group-interaction parameters are represented with filled symbols, and those not used are represented with open symbols. (a) Isobaric vapor–liquid equilibria of 5-methyloxolan-2-one + primary alcohols, with experimental data for methanol^95^ and ethanol.^95^ (b) Isobaric solid–liquid equilibria of oxepan-2-one + primary alcohols, with experimental data for methanol^66^ and propan-1-ol.^66^ (c) Density of oxolan-2-one + linear primary alcohols at 298.15 K, with experimental data for methanol,^97^ ethanol,^97^ butan-1-ol,^97^ and octan-1-ol.^97^ (d) Excess enthalpy of oxolan-2-one + primary alcohols at 298.15 K, with experimental data for methanol,^100^ hexan-1-ol,^96^ heptan-1-ol,^96^ octan-1-ol,^96^ and decan-1-ol.^96^ Thermodynamic conditions and the accuracy of the calculations are detailed in Table 9 and in the Zenodo datafile.

The unlike group interaction parameters are estimated from VLE,^92−95^ SLE,^66^ single-phase density,^96−98^ and excess enthalpy^99,100^ experimental data. In particular, the corresponding isobaric VLE data^95^ are shown in Figure 7a for 5-methyloxolan-2-one + methanol and 5-methyloxolan-2-ol + ethanol. No azeotrope is found in these phase diagrams. SLE data (Figure 7b) are available for mixtures of oxepan-2-one + methanol and oxepan-2-one + propan-1-ol; these are used to characterize the interaction parameters with more accuracy. Characteristic nonideal behavior is observed in the experimental density data^97,98^ of mixtures of oxolan-2-one and several linear primary alcohols (Figure 7c) at 298.15 K, with a concave shape for the shorter alcohols and a convex shape for the longer alcohols, as a function of mole fraction. The highest densities for *x*_oxolan-2-one_ < 0.1 are obtained for the longest chains (the longer/heavier alcohols), while the highest densities for *x*_oxolan-2-one_ > 0.1 are seen for the smallest chains. An inversion is observed for *x*_oxolan-2-one_ ≈ 0.1 in the mixtures considered, including the mixture of oxolan-2-one + methanol. Excess molar enthalpy data^99,100^ at 298.15 K and calculations are shown in Figure 7d. As can be seen, the excess molar enthalpies of the oxolan-2-one + long primary alcohol mixtures exhibit a similar trend and order of magnitude, with a maximum of approximately 2 kJ/mol at *x*_oxolan-2-one_ ≈ 0.5. The calculated excess enthalpy for oxolan-2-one + methanol presents a lower maximum; we note the limited experimental data available for this system.

**Figure 8:**
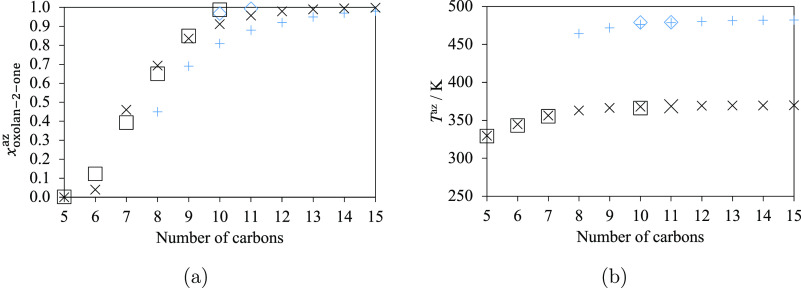
(a) Azeotrope compositions given as the mole fraction of oxolan-2-one (*x*_oxolan-2-one_^az^) and (b) azeotrope temperatures (*T*^az^) for mixtures of oxolan-2-one + linear primary alcohols. The number of carbons corresponds to the length of the linear primary alcohol (six for hexan-1-ol, seven for heptan-1-ol, etc.). Black “×” symbols and blue “+” symbols represent the calculations with SAFT-γ Mie. Black squares and blue diamonds represent the experimental points.^83^ Black and blue points correspond to results at 2.6664 and 101.3 kPa, respectively. Thermodynamic conditions and the accuracy of the calculations are detailed in Table 9 and in the Zenodo datafile.

Azeotrope experimental data^83^ for mixtures of oxolan-2-one and linear primary alcohols are also available (Figure 8) for mixtures containing linear primary alcohols from pentan-1-ol (C_5_H_11_OH) to undecan-1-ol (C_11_H_23_OH) but are not used for the parameter estimation. The azeotrope compositions *x*_oxolan-2-one_^az^ at 2666.4 Pa increase with an increase in the alcohol chain length (Figure 8a) and they are higher than 0.5 for oxolan-2-one + octan-1-ol (C_8_H_17_OH) and oxolan-2-one + longer alcohols. As noted earlier, the azeotrope temperatures also increase with an increase in the alkane chain length (Figure 8b) such that the results for the azeotrope compositions and temperatures are qualitatively similar for both oxolan-2-one + linear primary alcohols and oxolan-2-one + linear alkanes (Figure 6). We note, however, that the azeotrope compositions are higher in the mixtures with alcohols than in the mixtures with the alkanes for a given number of carbons in the chain.

Excellent agreement between the experimental data and the SAFT-γ Mie calculation is obtained for the VLE, with %AADs of 0.7520 and 0.1422% for the bubble temperatures and 0.4562 and 0.3411% for the dew temperatures of 5-methyloxolan-2-one + methanol and 5-methyloxolan-2-one + ethanol, respectively. The solubility of oxepan-2-one (i.e., the SLE curves for *T* > 200 K in Figure 7b) is determined with accuracy both in methanol (%AAD = 1.702%) and propan-1-ol (%AAD = 4.388%). Densities are also correctly described with SAFT-γ Mie. In particular, the characteristics of the curves shown in Figure 7c, for example, the concave/convex shapes and the intersection of the curves, are in excellent quantitative agreement with the experimental data. The highest %AAD is only 3.438% for oxolan-2-one + octan-1-ol. A concave shape is found for all of the excess enthalpy curves, as shown in Figure 7d. Good agreement is obtained with experiment,^99,100^ and all %AADs for excess enthalpy are smaller than 10% except for oxolan-2-one + decan-1-ol (11.06%). The prediction of the excess enthalpy of oxolan-2-one + methanol is significantly different in comparison with the other mixtures containing alcohol, as suggested by the experimental data (the predicted maximum is around 1 kJ/mol only).

The influence of the length of the alcohol chain on the VLE, SLE, densities, and excess enthalpies can also be gleaned from Figure 7 with additional predictions made for several mixtures for which no experimental data are available. We also predict the azeotrope composition and temperature with SAFT-γ Mie for mixtures of oxolan-2-one + linear primary alcohols until pentadecan-1-ol (C_15_H_31_OH). The overall predictions are in excellent agreement with the experiment (%AADs and AAD are detailed in Table 9). An azeotrope is mentioned in the experimental data^83^ for oxolan-2-one + pentan-1-ol at 2666.4 Pa with an unusually small composition (*x*_oxolan-2-one_^az,exp^ = 0.002) compared with the other data for the azeotropes considered here, while no azeotrope is predicted with SAFT-γ Mie for that mixture (which corresponds to %AAD = 100% and AAD = 0.002).

**Table 9:** Overview of the of Accuracy SAFT-γ Mie in the Calculation of Azeotrope Compositions *x*_oxolan-2-one_^az^ and Temperatures *T*^az^, Bubble Temperatures *T*_bub_, Dew Temperatures *T*_dew_, Bubble Pressures *P*_bub_, Solubilities *x*_1_^sat^, Densities ρ, and Molar Excess Enthalpies Δ*h*_mix_ for Mixtures of Lactones and Primary Alcohols, Where *N*_*s*,*p*_^D^ Is the Number of Experimental Data Used in the the Parameter Estimation, and *N*_*s*,*p*_^D,total^ Is the Number of Experimental Data Used to Calculate %AAD_*s*_*p* and AAD_*s*_*p* for System *s* and Property *p*

system *s* (1 + 2)	*T*/K range	*P*/MPa range	*x*_1_ range	*N*_*s*,*p*_^D^	*N*_*s*,*p*_^total^	%AAD_*s*_*x*_oxolan-2-one_^az^	AAD_*s*_*x*_oxolan-2-one_^az^	figure	ref
oxolan-2-one + pentan-1-ol	−	0.00267	−	0	1	100.0	0.002	8(a)	(83)
oxolan-2-one + hexan-1-ol	−	0.00267	−	0	1	67.48	0.083	8(a)	(83)
oxolan-2-one + heptan-1-ol	−	0.00267	−	0	1	17.30	0.06800	8(a)	(83)
oxolan-2-one + octan-1-ol	−	0.00267	−	0	1	6.716	0.04365	8(a)	(83)
oxolan-2-one + nonan-1-ol	−	0.00267	−	0	1	1.698	0.01444	8(a)	(83)
oxolan-2-one + decan-1-ol	−	0.00267–0.101	−	0	2	12.03	0.1173	8(a)	(83)
oxolan-2-one + undecan-1-ol	−	0.101	−	0	1	11.38	0.113	8(a)	(83)

system *s* (1 + 2)	*T*/K range	*P*/MPa range	*x*_1_ range	*N*_*s*,*p*_^D^	*N*_*s*,*p*_^total^	%AAD_*s*_*T*^az^	AAD_*s*_*T*^az^/K	figure	ref
oxolan-2-one + pentan-1-ol	−	0.00267	−	0	1	0.1874	0.6167	8(b)	(83)
oxolan-2-one + hexan-1-ol	−	0.00267	−	0	1	0.4756	1.632	8(b)	(83)
oxolan-2-one + heptan-1-ol	−	0.00267	−	0	1	0.3069	1.090	8(b)	(83)
oxolan-2-one + decan-1-ol	−	0.00267–0.101	−	0	2	0.5516	2.357	8(b)	(83)
oxolan-2-one + undecan-1-ol	−	0.101	−	0	1	0.0879	0.4210	8(b)	(83)

system *s* (1 + 2)	*T*/K range	*P*/MPa range	*x*_1_ range	*N*_*s*,*p*_^D^	*N*_*s*,*p*_^total^	%AAD_*s*_*T*_bub_	AAD_*s*_*T*_bub_/K	figure	ref
oxolan-2-one +1,4-butanediol	−	0.030–0.070	0.00–1.00	18	36	0.8126	3.792	−	(94)
5-methyloxolan-2-one + methanol	−	0.101	0.00–0.82	18	18	0.3492	1.269	7(a)	(95)
5-methyloxolan-2-one + ethanol	−	0.101	0.00–0.82	12	35	0.1422	0.5184	7(a)	(95)

system *s* (1 + 2)	*T*/K range	*P*/MPa range	*x*_1_ range	*N*_*s*,*p*_^D^	*N*_*s*,*p*_^total^	%AAD_*s*_*T*_dew_	AAD_*s*_*T*_dew_/K	figure	ref
oxolan-2-one +1,4-butanediol	−	0.030–0.070	0.00–1.00	18	36	0.8611	3.998	−	(94)
5-methyloxolan-2-one + methanol	−	0.101	0.00–0.03	6	18	0.3080	1.112	7(a)	(95)
5-methyloxolan-2-one + ethanol	−	0.101	0.00–0.05	12	35	0.3411	1.261	7(a)	(95)

system *s* (1 + 2)	*T*/K range	*P*/MPa range	*x*_1_ range	*N*_*s*,*p*_^D^	*N*_*s*,*p*_^total^	%AAD_*s*_*P*_bub_	AAD_*s*_*P*_bub_/Pa	figure	ref
oxolan-2-one +1,4-butanediol	373–423	−	0.00–1.00	32	32	14.73	795.3	−	(92,93)

system *s* (1 + 2)	*T*/K range	*P*/MPa range	*x*_1_ range	*N*_*s*,*p*_^D^	*N*_*s*,*p*_^total^	%AAD_*s*_*x*_1_^sat^	AAD_*s*_*x*_1_^sat^	figure	ref
oxepan-2-one in methanol	204–272	0.101	−	21	22	1.702	0.007757	7(b)	(66)
oxepan-2-one in propanol	204–272	0.101	−	19	20	4.388	0.01220	7(b)	(66)

system *s* (1 + 2)	*T*/K range	*P*/MPa range	*x*_1_ range	*N*_*s*,*p*_^D^	*N*_*s*,*p*_^total^	%AAD_*s*_ ρ	AAD_*s*_ ρ/(kg/m^3^)	figure	ref
oxolan-2-one + methanol	298	0.101	0.00–1.00	11	11	0.5953	6.281	7(c)	(97)
oxolan-2-one + ethanol	298	0.101	0.00–1.00	11	11	0.9588	8.877	7(c)	(97)
oxolan-2-one + butan-1-ol	293–303	0.101	0.00–1.00	24	59	1.209	11.29	7(c)	(97,98)
oxolan-2-one + octan-1-ol	298	0.101	0.00–1.00	0	11	3.438	31.92	7(c)	(97)
oxolan-2-one + 2-methyl-1-propanol	303	0.101	0.00–1.00	10	10	1.367	12.39	−	(96)
oxolan-2-one + 3-methyl-1-butanol	303	0.101	0.00–1.00	10	10	1.384	12.59	−	(96)

system *s* (1 + 2)	*T*/K range	*P*/MPa range	*x*_1_ range	*N*_*s*,*p*_^D^	*N*_*s*,*p*_^total^	%AAD_*s*_ Δ*h*_mix_	AAD_*s*_ Δ*h*_mix_/(J/mol)	figure	ref
oxolan-2-one + methanol	298	0.101	0.89–0.99	5	5	4.694	12.56	7(d)	(100)
oxolan-2-one + hexan-1-ol	298	0.101	0.12–0.94	12	12	7.594	113.3	7(d)	(99)
oxolan-2-one + heptan-1-ol	298	0.101	0.15–0.95	12	12	4.447	74.57	7(d)	(99)
oxolan-2-one + octan-1-ol	298	0.101	0.17–0.95	0	12	9.077	172.5	7(d)	(99)
oxolan-2-one + decan-1-ol	298	0.101	0.21–0.96	0	12	11.06	206.2	7(d)	(99)

Primary alcohols with a branched carbon chain are also considered in order to estimate the unlike interaction between the cCOO group and the CH group.^51^ Very good accuracy is obtained for the density of oxolan-2-one + 2-methyl-1-propanol (%AAD = 1.367%) and oxolan-2-one +3-methyl-1-butanol (%AAD = 1.384%). The corresponding unlike group parameters are detailed in Table 5, and the association parameters are listed in Table 6. The overall accuracy for all of these systems can be found in Table 9.

#### 3.2.2 Lactone + Secondary Alcohol Mixtures: cCOO–CHOH and cCH–CHOH Interactions

We now consider the modeling of lactones and secondary alcohols by incorporating the CHOH group,^11^ which is modeled with two sites of type e_1_ and one site of type H. The H site of the CHOH group interacts with the e_1_ sites of the cCOO group. The unlike group parameters characterized in this section are ε_cCOO–CHOH_,  and  (cf. Tables 5 and 6).

**Figure 9:**
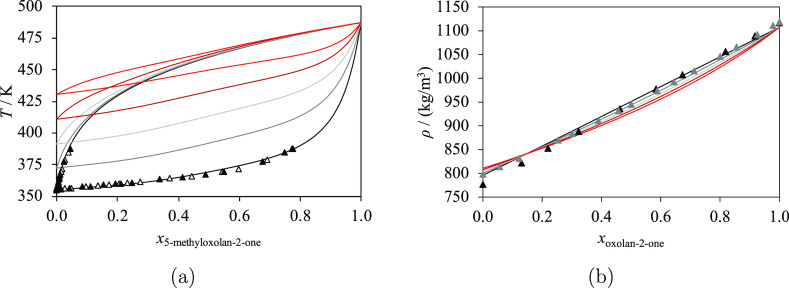
Selected isobaric thermodynamic properties of lactones + secondary alcohols: propan-2-ol (black), butan-2-ol (dark gray), pentan-2-ol (light gray), hexan-2-ol (dark red), and heptan-2-ol (red) at atmospheric pressure. The curves represent the calculations with SAFT-γ Mie. Experimental data used in the estimation of group-interaction parameters are represented with filled symbols, and those not used are represented with open symbols. (a) Isobaric vapor–liquid equilibria of 5-methyloxolan-2-one + secondary alcohols, with experimental data for propan-2-ol.^95^ (b) Density of oxolan-2-one + secondary alcohols at 303.15 K, with experimental data for propan-2-ol^96^ and butan-2-ol.^98^ Thermodynamic conditions and the accuracy of the calculations are detailed in Table 10 and in the Zenodo datafile.

Experimental VLE data,^95^ shown in Figure 9a, are available for 5-methyloxolan-2-one + propan-2-ol at atmospheric pressure for a large range of mole fractions for the bubble temperature (0.00 ≤ *x*_5-methyloxolan-2-one_ ≤ 0.77) and a small range for the dew temperature (0.00 ≤ *x*_5-methyloxolan-2-one_ ≤ 0.045). Experimental density data can be seen in Figure 9b for oxolan-2-one + propan-2-ol^96^ and oxolan-2-one + butan-2-ol.^98^ The accuracy for the description of mixtures of lactones and secondary alcohols is very good for both VLE and density. The corresponding %AAD is only 0.3007% for the bubble temperature of 5-methyloxolan-2-one + propan-2-ol, and 0.2153% for the related dew temperature. The overall agreement for the density is also very good and includes a crossing of curves at *x*_5-methyloxolan-2-one_ ∼ 0.15 seen both from experimental data and the SAFT-γ Mie calculations.

Predictions for the VLE of mixtures of 5-methyloxolan-2-one + butan-2-ol, + pentan-2-ol, + hexan-2-ol, and + heptan-2-ol are also shown in Figure 9a. No azeotrope is predicted for these systems at atmospheric pressure. Additional density predictions for oxolan-2-one + secondary alcohols are shown in Figure 9b, even if experimental data are not available for all the considered mixtures. The density is found to be more ideal for lactones + secondary alcohols than for lactones + primary alcohols (cf. Figure 7c). Other relevant information is summarized in Table 10.

**Table 10:** Overview of the Accuracy of SAFT-γ Mie in the Calculation of Bubble Temperatures *T*_bub_, Dew Temperatures *T*_dew_, and Densities ρ for Mixtures of Lactones and Secondary Alcohols, Where *N*_*s*,*p*_^D^ Is the Number of Experimental Data Used in the Parameter Estimation, and *N*_*s*,*p*_^D,total^ Is the Number of Experimental Data Used to Calculate %AAD_*s*_*p*and AAD_*s*_*p* for System *s* and Property *p*

system *s* (1 + 2)	*T*/K range	*P*/MPa range	*x*_1_ range	*N*_*s*,*p*_^D^	*N*_*s*,*p*_^total^	%AAD_*s*_*T*_bub_	AAD_*s*_*T*_bub_/K	figure	ref
5-methyloxolan-2-one + propan-2-ol	−	0.101	0.00–0.77	16	31	0.3007	1.094	9(a)	(95)

system *s* (1 + 2)	*T*/K range	*P*/MPa range	*x*_1_ range	*N*_*s*,*p*_^D^	*N*_*s*,*p*_^total^	%AAD_*s*_*T*_dew_	AAD_*s*_*T*_dew_/K	figure	ref
5-methyloxolan-2-one + propan-2-ol	−	0.101	0.00–0.045	8	31	0.2153	0.7907	9(a)	(95)

system *s* (1 + 2)	*T*/K range	*P*/MPa range	*x*_1_ range	*N*_*s*,*p*_^D^	*N*_*s*,*p*_^total^	%AAD_*s*_ ρ	AAD_*s*_ ρ/(kg/m^3^)	figure	ref
oxolan-2-one + propan-2-ol	303	0.101	0.00–1.00	10	10	0.8779	7.859	9(b)	(96)
oxolan-2-one + butan-2-ol	293–313	0.101	0.00–1.00	16	64	0.4341	4.501	9(b)	(98)

### 3.3 Mixtures of Lactones + 2-Ketones: cCOO–CH_3_COCH_3_ and cCOO–CH_3_CO Interactions

We now consider binary mixtures of lactones + 2-ketones, including acetone. SAFT-γ Mie models for acetone, as well as for 2-ketones, have been presented in previous work,^39,51^ and the same models are adopted here. Acetone is modeled as a single molecular group^51^ denoted by CH_3_COCH_3_, while other 2-ketones are modeled by using the CH_3_CO group^39^ together with CH_2_ and CH_3_ groups. No unlike association is considered between the cCOO group and ketone groups; thus, the only parameters that need to be estimated here are  and . Limited experimental data are available, which include VLE data^101^ at atmospheric pressure for 5-methyloxolan-2-one + acetone for a small range of *x*_5-methyloxolan-2-one_ values only and SLE data^66^ of oxepan-2-one + pentan-2-one with a eutectic point at 194.46 K. As can be seen in Table 11, most of the experimental data available are used to characterize the parameters.

**Table 11:** Overview of the Accuracy of SAFT-γ Mie in the Calculation of Bubble Temperatures *T*_bub_, Dew Temperatures *T*_dew_, and Solubilities *x*_1_^sat^ for Binary Mixtures of Lactones + 2-Ketones, Where *N*_*s*,*p*_^D^ Is the Number of Experimental Data Used in Parameter Estimation, and *N*_*s*,*p*_^D,total^ is the Number of Experimental Data Used to Calculate %AAD_*s,p*_ and AAD_*s,p*_ for System *s* and Property *p*

system *s* (1 + 2)	*T*/K range	*P*/MPa range	*x*_1_ range	*N*_*s*,*p*_^D^	*N*_*s*,*p*_^total^	%AAD_*s*_*T*_bub_	AAD_*s*_*T*_bub_/K	figure	ref
5-methyloxolan-2-one + acetone	−	0.101	0.00–0.42	30	30	0.02819	0.09421	10(a)	(101)

system *s* (1 + 2)	*T*/K range	*P*/MPa range	*x*_1_ range	*N*_*s*,*p*_^D^	*N*_*s*,*p*_^total^	%AAD_*s*_*T*_dew_	AAD_*s*_*T*_dew_/K	figure	ref
5-methyloxolan-2-one + acetone	−	0.101	0–0.0062	15	30	1.300	4.360	10(a)	(101)

system *s* (1 + 2)	*T*/K range	*P*/MPa range	*x*_1_ range	*N*_*s*,*p*_^D^	*N*_*s*,*p*_^total^	%AAD_*s*_*x*_1_^sat^	AAD_*s*_*x*_1_^sat^	figure	ref
oxepan-2-one in pentan-2-one	194–272	0.101	−	19	21	3.704	0.01204	10(b)	(66)

**Figure 10:**
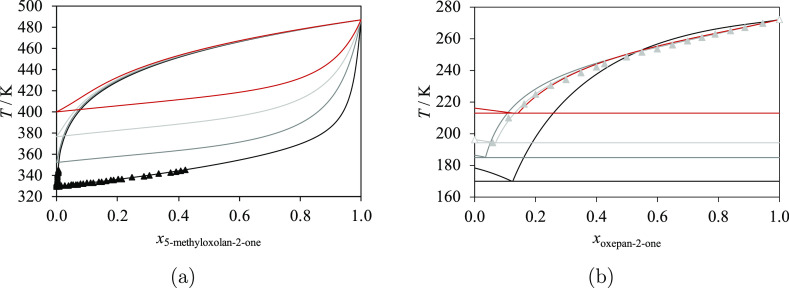
Isobaric phase diagrams of lactones + 2-ketones: acetone (black), butan-2-one (dark gray), pentan-2-one (light gray), hexan-2-one (dark red), heptan-2-one (red), and octan-2-one (orange) at atmospheric pressure. The curves represent the calculations with SAFT-γ Mie. Experimental data used in the estimation of group-interaction parameters are represented with filled symbols, and those not used are represented with open symbols. (a) Vapor–liquid equilibria of 5-methyloxolan-2-one + 2-ketones, with experimental data for acetone.^101^ (b) Solid–liquid equilibria of oxepan-2-one + 2-ketones, with experimental data for pentan-2-one.^66^ Thermodynamic conditions and accuracy are detailed in Table 11 and in the Zenodo datafile.

Both the VLE of 5-methyloxolan-2-one + acetone and the SLE of oxepan-2-one + pentan-2-one are accurately described with the resulting SAFT-γ Mie model (Figure 10). The corresponding AADs are small, as are the %AADs, which are below 4% for all the available properties (Table 11). Additional VLE diagrams at atmospheric pressure are predicted for binary mixtures of 5-methyloxolan-2-one + butan-2-one, pentan-2-one, and hexan-2-one. No azeotrope is predicted for these systems. The SLE phase diagrams of oxepan-2-one + acetone, + butan-2-one, and + hexan-2-one are also predicted. All of these systems have a eutectic point, as can be seen for oxepan-2-one + pentan-2-one both from experiments and the SAFT calculations. These VLE and SLE predictions can also be seen in Figure 10.

### 3.4 Mixtures of Lactones + Linear Esters: cCOO–COO Interactions

**Figure 11:**
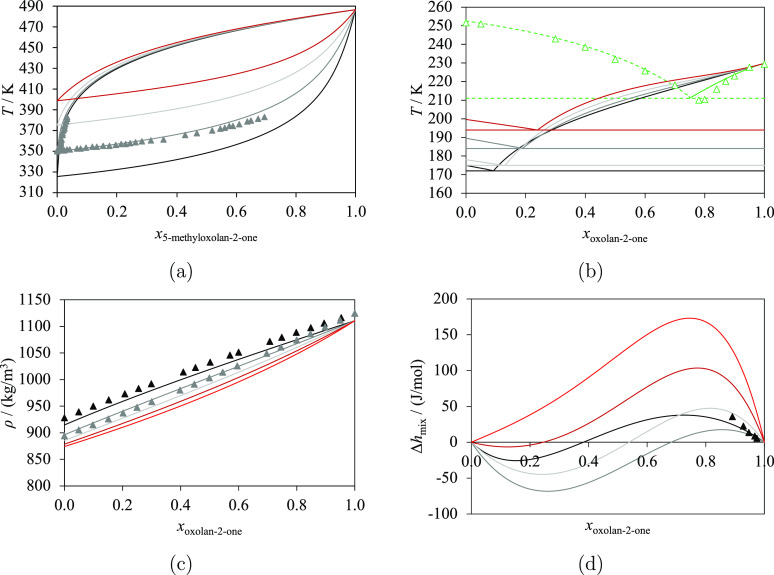
Isobaric thermodynamic properties of lactones + linear esters at atmospheric pressure: methyl acetate (black), ethyl acetate (dark gray), propyl acetate (light gray), butyl acetate (dark red), pentyl acetate (red), and diethylbutanedioate (green). The curves represent the calculations with SAFT-γ Mie. The dashed curves are determined with Δ*h*_diethylbutanedioate_^fus, estimated^, as explained in the text. Experimental data used in the estimation of group-interaction parameters are represented with filled symbols, and those not used are represented with open symbols. (a) Vapor–liquid equilibria of 5-methyloxolan-2-one + esters, with experimental data for ethyl acetate.^101^ (b) Solid–liquid equilibria of oxolan-2-one + esters, with experimental data for diethylbutanedioate.^70^ (c) Density of oxolan-2-one + esters at 298.15 K, with experimental data for methyl acetate^102^ and ethyl acetate.^102^ (d) Excess molar enthalpy of oxolan-2-one + esters at 298.15 K, with experimental data for methyl acetate.^103^ Thermodynamic conditions and the accuracy of the calculations are detailed in Table 12 and in the Zenodo datafile.

Experimental data of mixtures of lactones and linear esters are available for several properties, including VLE,^70,101^ SLE,^70^ density,^102^ and molar excess enthalpy^103^ (see Figure 11). In particular, VLE data at atmospheric pressure are available for the mixture of 5-methyloxolan-2-one + ethyl acetate.^101^ SLE data for oxolan-2-one + diethylbutanedioate^70^ are also available, with a eutectic point at *x*_oxolan-2-one_ ≈ 0.78 and *T*_eutectic_ ≈ 210 K. Density data at 298.15 K and 101.3 kPa are available for oxolan-2-one + methyl acetate and oxolan-2-one + ethyl acetate^102^ for *x*_oxolan-2-one_ ∈ [0.0, 1.0]. Excess molar enthalpy data at 298.15 K and 101.3 kPa are available for oxolan-2-one + methyl acetate^103^ for the small range *x*_oxolan-2-one_ ∈ [0.89, 0.97] with the largest value of only 35.7 J/mol (for *x*_oxolan-2-one_ = 0.89).

The characterization of the interaction parameters between the cCOO and COO^8^ groups in the SAFT-γ Mie modeling is performed by considering the VLE, density, and excess enthalpy data available. SLE data are not used to estimate the parameters here. The resulting agreement between the calculations and the experimental data is excellent for all of the properties. In particular, no azeotrope is found in the VLE of 5-methyloxolan-2-one + ethyl acetate at atmospheric pressure. The density at 298.15 K is larger for oxolan-2-one + methyl acetate than for oxolan-2-one + ethyl acetate, for all values of *x*_oxolan-2-one_, which is qualitatively consistent with the experimental findings. The agreement for the excess molar enthalpy of oxolan-2-one + methyl acetate is also very good despite the small values found for this property (quantitatively, we obtain %AAD = 16.86%, which corresponds to an AAD of 3.536 J/mol).

The resulting group parameters are used to make additional predictions of the VLE, SLE, density, and excess molar enthalpy for binary mixtures of lactones and methyl, ethyl, propyl, and pentyl acetates; these are shown in Figure 11. It is of interest to note the “S” shape predicted for the molar excess enthalpy curves shown in Figure 11d, with positive values for lactone-rich phases and negative values for ester-rich phases. For oxolan-2-one + pentyl acetate, the predicted excess enthalpy is always positive, although highly asymmetric. More experimental data would be useful to confirm or disprove these predictions.

The SLE of oxolan-2-one + diethylbutanedioate for *x*_oxolan-2-one_ > 0.78 is correctly predicted by using the melting temperature and the enthalpy of fusion of oxolan-2-one^56^ (cf. Table 1). The melting temperature of diethylbutanedioate^61^ is reported as *T*_diethylbutanedioate_^fus^ = 252.55 K (cf. Table 1); however, to the best of our knowledge, there is no experimental value of the corresponding enthalpy of fusion. The %AAD for the SLE shown in Figure 11b as a dashed curve for *x*_oxolan-2-one_ < 0.78 can be minimized by estimating the value of the enthalpy of fusion, which yields Δ*h*_diethylbutanedioate_^fus, estimated^ = 23 kJ/mol (such that %AAD = 6.238% and AAD = 0.01237). The value of Δ*h*_diethylbutanedioate_^fus, estimated^ is close to the value obtained from the Joback group-contribution method:^104^ Δ*h*_diethylbutanedioate_^fus, Joback^ = 20.03 kJ/mol (we note that both values are higher than the values reported in Table 1 for other compounds). The eutectic composition and temperature are also correctly predicted, as *x*_oxolan-2-one_^predicted^ ∼ 0.75, and *T*_eutectic_^predicted^ ∼ 211 K with the estimated enthalpy of fusion. Additional information about the accuracy of the SAFT-γ Mie approach for these systems is summarized in Table 12.

**Table 12:** Overview of the Accuracy of SAFT-γ Mie in the Calculation of Bubble Temperatures *T*_bub_, Dew Temperatures *T*_dew_, Solubilities *x*_1_^sat^, Densities ρ, and Excess Molar Enthalpies Δ*h*_mix_ for Binary Mixtures of Lactones + Esters, Where *N*_*s*,*p*_^D^ Is the Number of Experimental Data Used in the Parameter Estimation, and *N*_*s*,*p*_^D,total^ Is the Number of Experimental Data Used to Calculate %AAD_*s*_*p* and AAD_*s*_*p* for System *s* and Property *p*

system *s* (1 + 2)	*T*/K range	*P*/MPa range	*x*_1_ range	*N*_*s*,*p*_^D^	*N*_*s*,*p*_^total^	%AAD_*s*_*T*_bub_	AAD_*s*_*T*_bub_/K	figure	ref
oxolan-2-one + diethyl butanedioate	−	0.012	0.00–1.00	21	24	2.152	8.871	−	(70)
5-methyloxolan-2-one + ethyl acetate	−	0.101	0.00–0.69	36	36	0.5151	1.917	11(a)	(101)

system *s* (1 + 2)	*T*/K range	*P*/MPa range	*x*_1_ range	*N*_*s*,*p*_^D^	*N*_*s*,*p*_^total^	%AAD_*s*_*T*_dew_	AAD_*s*_*T*_dew_/K	figure	ref
oxolan-2-one + diethyl butanedioate	−	0.012	0.00–1.00	21	24	2.362	9.723	−	(70)
5-methyloxolan-2-one + ethyl acetate	−	0.101	0.00–0.03	36	36	2.495	9.005	11(a)	(101)

system *s* (1 + 2)	*T*/K range	*P*/MPa range	*x*_1_ range	*N*_*s*,*p*_^D^	*N*_*s*,*p*_^total^	%AAD_*s*_*x*_1_^sat^	AAD_*s*_*x*_1_^sat^	figure	ref
oxolan-2-one in diethyl butanedioate	210–252	0.101	−	0	8	6.238	0.01237	11(b)	(70)
diethyl butanedioate in oxolan-2-one	210–229	0.101	−	0	7	3.099	0.02592	11(b)	(70)

system *s* (1 + 2)	*T*/K range	*P*/MPa range	*x*_1_ range	*N*_*s*,*p*_^D^	*N*_*s*,*p*_^total^	%AAD_*s*_ ρ	AAD_*s*_ ρ/(kg/m^3^)	figure	ref
oxolan-2-one + methyl acetate	298	0.101	0.00–1.00	19	19	1.312	13.50	11(c)	(102)
oxolan-2-one + ethyl acetate	298	0.101	0.00–1.00	19	19	0.4436	4.634	11(c)	(102)

system *s* (1 + 2)	*T*/K range	*P*/MPa range	*x*_1_ range	*N*_*s*,*p*_^D^	*N*_*s*,*p*_^total^	%AAD_*s*_ Δ*h*_mix_	Δ*h*_mix_/(J/mol)	figure	ref
oxolan-2-one + methyl acetate	298	0.101	0.89–0.97	5	5	16.86	3.536	11(d)	(103)

### 3.5 Mixtures of Lactones + Aromatic Compounds: cCOO–aCH, cCOO–aCCH_3_, and cCOO–aCCH_2_ Interactions

The aromatic compounds are modeled with specific aromatic groups, denoted as aCH, aCCH_3_, and aCCH_2_. For instance, benzene is composed of six aCH groups.^51^ One of the aCH groups of benzene is replaced by an aCCH_3_ group^37,90^ to model toluene or replaced by an aCCH_2_ group^51^ bonded to a CH_3_ group to model ethylbenzene. There is no association between the cCOO group and the aromatic groups, such that only the unlike interaction parameters ε_*kl*_ and λ_*kl*_^r^ need to be characterized (Table 5) to describe binary mixtures of lactones + aromatic compounds.

**Figure 12:**
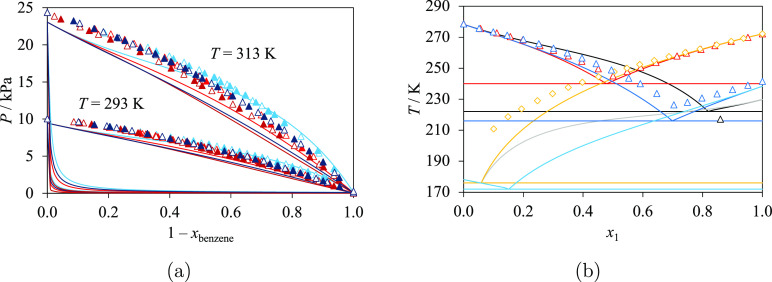
Phase diagrams of lactones + aromatic compounds. The curves represent the calculations with SAFT-γ Mie. Experimental data used in the estimation of group-interaction parameters are represented with filled symbols, and those not used are represented with open symbols. (a) Isothermal vapor–liquid equilibria of lactones + benzene: oxolan-2-one (light blue), oxan-2-one (red), oxepan-2-one (dark red), and 5-methyloxolan-2-one (dark blue) at 293 and 313 K, with experimental data for oxolan-2-one,^105^ oxan-2-one,^105^ oxepan-2-one,^105^ and 5-methyloxolan-2-one.^105^ (b) Isobaric solid–liquid equilibria at atmospheric pressure of compounds (1) + (2): oxolan-2-one + benzene (black), oxolan-2-one + toluene (gray), oxepan-2-one + benzene (red), oxepan-2-one + toluene (yellow), 5-methyloxolan-2-one + benzene (dark blue), and 5-methyloxolan-2-one + toluene (light blue), with experimental data for oxolan-2-one + benzene,^108^ oxepan-2-one + benzene,^66^ oxepan-2-one + toluene,^66^ and 5-methyloxolan-2-one + benzene.^109^ Thermodynamic conditions and the accuracy of the calculations are detailed in Table 13 and in the Zenodo datafile.

**Figure 13:**
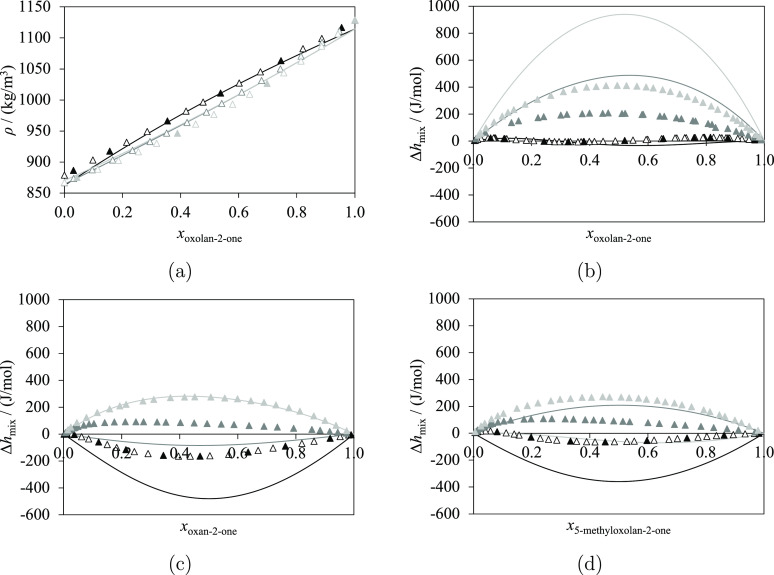
Isobaric thermodynamic properties of binary mixtures of lactone + aromatic compound: benzene (black), toluene (dark gray), and ethylbenzene (light gray) at atmospheric pressure. The curves represent calculations with SAFT-γ Mie. Experimental data used in the estimation of group-interaction parameters are represented with filled symbols, and those not used are represented with open symbols. (a) Density of oxolan-2-one + aromatic compounds at 293.15 K, with experimental data for benzene,^106^ toluene,^106^ and ethylbenzene.^106^ (b) Excess molar enthalpy of oxolan-2-one + aromatic compounds at 293.15 K, with experimental data for benzene,^105^ toluene,^105^ and ethylbenzene.^105^ (c) Excess enthalpy of oxan-2-one + aromatic compounds at 293.15 K, with experimental data for benzene,^107^ toluene,^107^ and ethylbenzene.^107^ (d) Excess enthalpy of 5-methyloxolan-2-one + aromatic compounds at 293.15 K, with experimental data for benzene,^107^ toluene,^107^ and ethylbenzene.^107^ Thermodynamic conditions and the accuracy of the calculations are detailed in Table 13 and in the Zenodo datafile.

Bubble-pressure data^105^ at 293 and 313 K are used in the characterization of the unlike parameters considering mixtures of benzene + oxolan-2-one, + oxan-2-one, + oxepan-2-one, and + 5-methyloxolan-2-one. The VLE of these mixtures can be seen in Figure 12a for a given temperature; the markedly different vapor pressures of the pure lactones (some hundreds of Pa, as shown in Figures 2–4) and pure benzene^105^ (10.03 kPa at 293 K and 24.38 kPa at 313 K) give rise to clear nonideality in the phase diagrams. We also use density data^106^ for mixtures of oxolan-2-one + benzene, + toluene, and + ethylbenzene to characterize the unlike parameters (cf. Figure 13a). Excess molar enthalphy data,^105,107^ shown in Figure 13b–d, represent the most abundant set of data for the mixtures of lactones (oxolan-2-one, oxan-2-one, and 5-methyloxolan-2-one) and aromatic compounds (benzene, toluene, and ethylbenzene). The reported values are positive, as well as negative, depending on the mixture and are remarkably small with a maximum value of only 413 J/mol for an equimolar mixture of oxolan-2-one + ethylbenzene. The shape of some of the excess-enthalpy curves is also unusual; for example, the excess enthalpy of oxolan-2-one + benzene has an “M” shape^105^ (cf. Figure 13b) with positive values for *x*_oxolan-2-one_ < 0.21 and *x*_oxolan-2-one_ > 0.48, negative values otherwise, and a maximum of about 30 J/mol. SLE data are also available for mixtures of lactones and aromatic compounds^66,108,109^ but are not used in the characterization of group interactions.

The SAFT-γ Mie calculations using the optimized parameters are also shown in Figures 12 and 13. While the agreement for the bubble-pressure and density curves is excellent, the agreement for the excess enthalpy curves is not always quantitatively accurate. The sign is not correct for two of the nine mixtures considered (for oxan-2-one + toluene and 5-methyloxolan-2-one + ethylbenzene), although we note that the highest values obtained from the calculations and the experimental data are always of the order of a few hundred J/mol only. Encouragingly, the “M” shape of the excess enthalpy curve for oxolan-2-one + benzene is qualitatively obtained with SAFT.

The transferability of the parameters characterized is assessed for the prediction of the SLE (Figure 12b). As can be seen in the figure, the eutectic points of oxolan-2-one + benzene and 5-methyloxolan-2-one + benzene are correctly predicted. The corresponding %AADs and AAD for the systems^66,105−109^ shown in Figures 12 and 13 (and additional systems^110−113^) can be found in Table 13.

**Table 13:** Overview of the Accuracy of SAFT-γ Mie in the Calculation of Bubble Pressures *P*_bub_, Solubilities *x*_1_^sat^, Densities ρ, and Excess Enthalpies Δ*h*_mix_ for Binary Mixtures of Lactones + Aromatic Compounds, Where *N*_*s*,*p*_^D^ Is the Number of Experimental Data Used in the Parameter Estimation, and *N*_*s*,*p*_^D,total^ Is the Number of Experimental Data Used to Calculate %AAD_*s*_*p* and AAD_*s*_*p* for System *s* and Property *p*

system *s* (1 + 2)	*T*/K range	*P*/MPa range	*x*_1_ range	*N*_*s*,*p*_^D^	*N*_*s*,*p*_^total^	%AAD_*s*_*P*_bub_	AAD_*s*_*P*_bub_/Pa	figure	ref
oxolan-2-one + benzene	292–313	−	0.00–1.00	31	65	5.442	534.9	12(a)	(105)
oxan-2-one + benzene	292–313	−	0.00–1.00	32	68	14.17	1093	12(a)	(105)
oxepan-2-one + benzene	292–313	−	0.00–1.00	33	70	20.81	1327	12(a)	(105)
5-methyloxolan-2-one + benzene	292–313	−	0.00–1.00	37	77	22.81	1793	12(a)	(105)

system *s* (1 + 2)	*T*/K range	*P*/MPa range	*x*_1_ range	*N*_*s*,*p*_^D^	*N*_*s*,*p*_^total^	%AAD_*s*_*x*_1_^sat^	AAD_*s*_*x*_1_^sat^	figure	ref
oxolan-2-one in benzene	217	0.101	−	0	1	20.75	0.1784	12(b)	(108)
oxepan-2-one in benzene	248–272	0.101	−	0	10	1.671	0.01045	12(b)	(66)
benzene in oxepan-2-one	245–279	0.101	−	0	11	6.771	0.04391	12(b)	(66)
oxepan-2-one in toluene	211–272	0.101	−	0	19	20.99	0.05076	12(b)	(66)
5-methyloxolan-2-one in benzene	226–241	0.101	−	0	7	12.01	0.09369	12(b)	(109)
benzene in 5-methyloxolan-2-one	234–279	0.101	−	0	14	10.72	0.05832	12(b)	(109)

system *s* (1 + 2)	*T*/K range	*P*/MPa range	*x*_1_ range	*N*_*s*,*p*_^D^	*N*_*s*,*p*_^total^	%AAD_*s*_ ρ	AAD_*s*_ ρ/(kg/m^3^)	figure	ref
oxolan-2-one + benzene	293–343	0.101	0.00–1.00	32	102	0.6534	6.233	13(a)	(106)
oxolan-2-one + toluene	293–353	0.101	0.00–1.00	0	119	0.4534	4.454	13(a)	(106)
oxolan-2-one +1,2-dimethylbenzene	293–308	0.101	0.00–1.00	0	44	1.183	11.55	−	(110)
oxolan-2-one +1,4-dimethylbenzene	293–308	0.101	0.00–1.00	0	44	0.4880	4.829	−	(111)
oxolan-2-one +1,2,4-trimethylbenzene	288–308	0.101	0.00–1.00	0	85	0.7613	7.468	−	(112)
oxolan-2-one +1,3,5-trimethylbenzene	288–308	0.101	0.00–1.00	0	85	0.3751	3.721	−	(112)
oxolan-2-one + ethylbenzene	293–353	0.101	0.00–1.00	24	119	1.152	10.88	13(a)	(106)
5-methyloxolan-2-one + benzene	298	0.101	0.0008–0.006	0	5	1.814	15.86	−	(113)

system *s* (1 + 2)	*T*/K range	*P*/MPa range	*x*_1_ range	*N*_*s*,*p*_^D^	*N*_*s*,*p*_^total^	%AAD_*s*_ Δ*h*_mix_	AAD_*s*_ Δ*h*_mix_/(J/mol)	figure	ref
oxolan-2-one + benzene	293	0.101	0.0036–0.97	12	56	286.9	22.58	13(b)	(105)
oxolan-2-one + toluene	293	0.101	0.0082–0.99	31	31	129.3	183.2	13(b)	(105)
oxolan-2-one + ethylbenzene	293	0.101	0.017–0.98	36	36	116.7	314.0	13(b)	(105)
oxan-2-one + benzene	293	0.101	0.0021–0.99	11	31	1741	159.1	13(c)	(107)
oxan-2-one + toluene	293	0.101	0.0068–0.97	32	32	166.7	99.55	13(c)	(107)
oxan-2-one + ethylbenzene	293	0.101	0.015–0.98	24	25	7.572	9.043	13(c)	(107)
5-methyloxolan-2-one + benzene	293	0.101	0.0047–0.98	9	35	1780	390.8	13(d)	(107)
5-methyloxolan-2-one + toluene	293	0.101	0.010–0.97	31	31	444.7	318.6	13(d)	(107)
5-methyloxolan-2-one + ethylbenzene	293	0.101	0.0054–0.98	33	33	141.4	259.8	13(d)	(107)

### 3.6 Mixtures of Lactones + Water: cCOO–H_2_O Interactions

In the SAFT-γ Mie approach, water is modeled by an H_2_O molecular group^9,10^ that includes two association sites of type e_1_ (one for each lone pair of electrons of the oxygen atom) and two sites of type H (corresponding to the hydrogen atoms). The H_2_O–H_2_O e_1_–H interactions are incorporated into the pure-water model. In mixtures with lactones, hydrogen bonding interactions between the H sites in the H_2_O group and the e_1_ sites of the cCOO group are also accounted for. The cCOO–H_2_O unlike group interaction, thus, includes three parameters (, , and ); these are characterized in this section.

**Figure 14:**
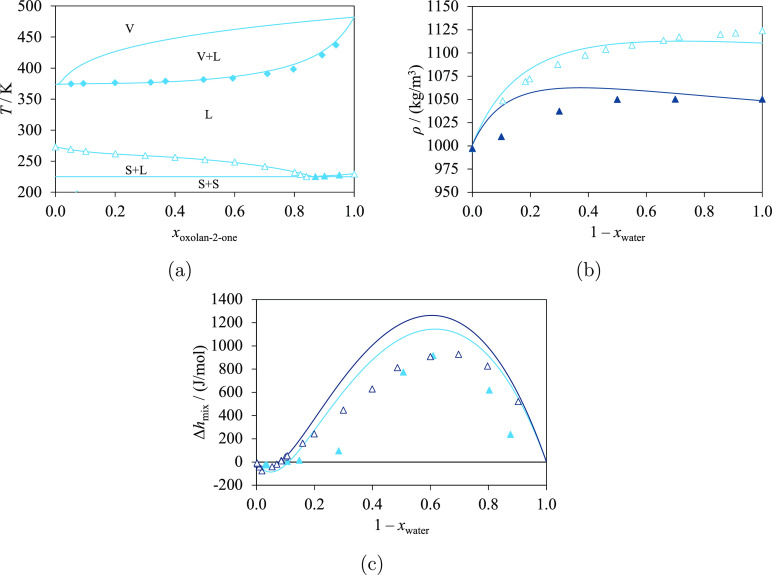
Isobaric thermodynamic properties of lactones + water at atmospheric pressure: oxolan-2-one (light blue) and 5-methyloxolan-2-one (dark blue). The curves represent the calculations with SAFT-γ Mie. Experimental data used in the estimation of group-interaction parameters are represented with filled symbols, and those not used are represented with open symbols. (a) Isobaric vapor–liquid equilibrium and solid–liquid equilibrium of oxolan-2-one + water, with experimental data for the bubble temperature^114^ and solid–liquid equilibrium.^70^ (b) Density of lactones + water at 298.15 K, with experimental data for oxolan-2-one^116^ and 5-methyloxolan-2-one.^117^ (c) Excess molar enthalpy of oxolan-2-one + water at 299.15 K and 5-methyloxolan-2-one + water at 303.15 K, with experimental data for oxolan-2-one + water^118^ and 5-methyloxolan-2-one + water.^73^ Thermodynamic conditions and the accuracy of the calculations are detailed in Table 14 and in the Zenodo datafile.

The VLE^114^ and SLE^70^ experimental data found for oxolan-2-one + water are represented in Figure 14a. No azeotropic or liquid–liquid demixing behavior is seen, but we note the eutectic point in the SLE region. Densities^115−117^ at 298.15 K and atmospheric pressure are presented in Figure 14b for binary mixtures of oxolan-2-one + water and 5-methyloxolan-2-one + water, and the excess molar enthalpies^73,118^ for the same mixtures are shown in Figure 14c. A highly asymmetric “S” shape can be seen for the two curves, with positive excess enthalpies for *x*_water_ < 0.9 and small negative values for *x*_water_ > 0.9. The highest values are about 1 kJ/mol for *x*_water_ ≈ 0.4 for the two systems. As can be gleaned from the figures, very good agreement is obtained for the bubble temperature of oxolan-2-one + water (%AAD = 0.7953%, and AAD = 2.978 K), the solubility of oxolan-2-one in water (%AAD = 0.3427%, and AAD = 0.003086), and the solubility of water in oxolan-2-one (%AAD = 3.168%, and AAD = 0.01049). The experimental^70^ eutectic composition and temperature are also accurately predicted. The calculated densities decrease with an increase in the length of the lactone side chain, and the agreement with experimental data^115−117^ is very good for water + oxolan-2-one and reasonable for water +5-methyloxolan-2-one. The corresponding %AADs and AADs are found to be small (cf. Table 14). Furthermore, the “S” shape of the excess enthalpy curves is correctly reproduced by the SAFT-γ Mie calculation. The corresponding %AADs are rather large (251.2% for water + oxolan-2-one, and 31.32% for water + 5-methyloxolan-2-one), although we note that these large values are due to the very small enthalpies in the water-rich phase (we have considered all the experimental data^73,118,119^ found for the %AAD calculation and not only the points shown in Figure 14c, including data for different temperatures and data at infinite dilution). The corresponding AADs, which are relatively small (43.33 J/mol for water + oxolan-2-one and 129.4 J/mol for water + 5-methyloxolan-2-one), provide a more appropriate measure of the quality of the model and confirm the good agreement seen in Figure 14c. Additional information on the accuracy of the calculations for the systems considered in this section compared with experimental data from the literature^70,73,114−122^ can be found in Table 14.

**Table 14:** Overview of the Accuracy of SAFT-γ Mie in the Calculation of Bubble Temperatures *T*_bub_, Dew Temperatures *T*_dew_, Liquid–Liquid Equilibrium Compositions *x*_1_^LLE^, Solubilities *x*_1_^sat^, Densities ρ, and Excess Molar Enthalpies Δ*h*_mix_ for Binary Mixtures of Lactones + Water, Where *N*_*s*,*p*_^D^ Is the number of Experimental Data Used in the Parameter Estimation, and *N*_*s*,*p*_^D,total^ Is the Number of Experimental Data Used to Calculate %AAD_*s*_*p* and AAD_*s*_*p* for System *s* and Property *p*

system *s* (1 + 2)	*T*/K range	*P*/MPa range	*x*_1_ range	*N*_*s*,*p*_^D^	*N*_*s*,*p*_^total^	%AAD_*s*_*T*_bub_	AAD_*s*_*T*_bub_/K	figure	ref
oxolan-2-one + water	−	0.012–0.101	0.02–1.00	11	76	0.7953	2.978	14(a)	(70,114,120)
5-methyloxolan-2-one + water	−	0.010–0.101	0.00–1.00	8	42	0.3850	1.456	−	(121)

system *s* (1 + 2)	*T*/K range	*P*/MPa range	*x*_1_ range	*N*_*s*,*p*_^D^	*N*_*s*,*p*_^total^	%AAD_*s*_*T*_dew_	AAD_*s*_*T*_dew_/K	figure	ref
oxolan-2-one + water	−	0.012–0.080	0.009–1.00	0	65	0.9717	3.623	14(a)	(70,120)
5-methyloxolan-2-one + water	−	0.010–0.101	0.00–1.00	0	42	0.8737	3.171	−	(121)

system *s* (1 + 2)	*T*/K range	*P*/MPa range	*x*_1_ range	*N*_*s*,*p*_^D^	*N*_*s*,*p*_^total^	%AAD_*s*_*x*_1_^LLE^	AAD_*s*_*x*_1_^LLE^	figure	ref
5-pentyloxolan-2-one + water	323–373	0.101	−	6	6	89.81	0.0009193	−	(122)

system *s* (1 + 2)	*T*/K range	*P*/MPa range	*x*_1_ range	*N*_*s*,*p*_^D^	*N*_*s*,*p*_^total^	%AAD_*s*_*x*_1_^sat^	AAD_*s*_*x*_1_^sat^	figure	ref
oxolan-2-one in water	225–229	0.101	−	3	4	0.3427	0.003086	14(a)	(70)
water in oxolan-2-one	225–273	0.101	−	0	12	3.168	0.01049	14(a)	(70)

system *s* (1 + 2)	*T*/K range	*P*/MPa range	*x*_1_ range	*N*_*s*,*p*_^D^	*N*_*s*,*p*_^total^	%AAD_*s*_ ρ	AAD_*s*_ ρ/(kg/m^3^)	figure	ref
oxolan-2-one + water	298–343	0.101	0.00–1.00	0	43	0.6222	6.664	14(b)	(115,116)
5-methyloxolan-2-one + water	298–323	0.101	0.00–1.00	12	12	1.338	13.63	14(b)	(117)
5-ethyloxolan-2-one + water	288–318	0.101	0.00081–0.0048	8	48	0.3988	3.983	−	(119)
6-methyloxan-2-one + water	288–318	0.101	0.0012–0.0050	10	39	0.3811	3.806	−	(119)

system *s* (1 + 2)	*T*/K range	*P*/MPa range	*x*_1_ range	*N*_*s*,*p*_^D^	*N*_*s*,*p*_^total^	%AAD_*s*_ Δ*h*_mix_	AAD_*s*_ Δ*h*_mix_/(J/mol)	figure	ref
oxolan-2-one + water	288–318	0.101	0.0012–0.88	7	51	251.2	43.33	14(c)	(118,119)
5-methyloxolan-2-one + water	303–323	0.101	0.0019–0.90	14	28	31.32	129.4	14(c)	(73)
5-ethyloxolan-2-one + water	288–318	0.101	0.00081–0.0048	12	48	49.08	3.420	−	(119)
6-methyloxan-2-one + water	288–318	0.101	0.0012–0.0050	10	39	58.61	11.35	−	(119)

### 3.7 Mixtures of Lactones + Carbon Dioxide: cCOO–CO_2_ Interactions

Carbon dioxide is modeled with the CO_2_ molecular group in SAFT-γ Mie.^90^ One association site of type e_1_ and one of e_2_ are incorporated in this group, and these are active in mixtures of CO_2_ with water or amines;^13,42^ they are not, however, assumed to interact with the e_1_ sites of the cCOO lactone group. Thus, the only unlike group parameters that need to be determined to characterize the cCOO–CO_2_ interactions are  and .

**Figure 15:**
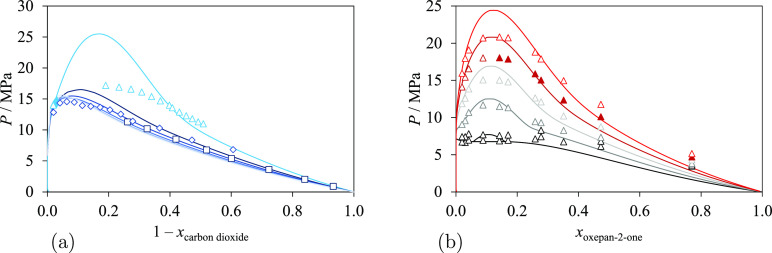
Isothermal bubble and dew pressures of lactone + carbon dioxide binary mixtures. The curves represent the calculations with SAFT-γ Mie. Experimental data used in the estimation of group-interaction parameters are represented with filled symbols, and those not used are represented with open symbols. (a) Fluid−phase equilibria of lactones + carbon dioxide: oxolan-2-one (light blue), 5-methyloxolan-2-one, 5-ethyloxolan-2-one, 5-propyloxolan-2-one, 5-butyloxolan-2-one, 5-pentyloxolan-2-one, and 5-hexyloxolan-2-one (from dark blue to pale blue) at 333 K. Experimental data are represented by triangles for oxolan-2-one + carbon dioxide,^124^ diamonds for 5-methyloxolan-2-one + carbon dioxide,^126^ and squares for 5-ethyloxolan-2-one + carbon dioxide.^125^ (b) Fluid−phase equilibria of oxepan-2-one + carbon dioxide at 303 K (black), 313 K (dark gray), 323 K (light gray), 333 K (dark red), and 343 K (red). Experimental data are represented by triangles.^125^ Thermodynamic conditions and the accuracy of the calculations are detailed in Table 15 and in the Zenodo datafile.

Experimental-bubble pressure data for mixtures of lactones + carbon dioxide are available,^123−126^ although we note that inconsistencies have been reported for some of the data related to these mixtures.^125^ We use the most recent set of data^125^ to estimate the cCOO–CO_2_ interaction parameters and present a representative sample of the results in Figure 15. Bubble pressures at 333 K for several binary lactone + carbon dioxide mixtures are shown in Figure 15a, and the influence of temperature is shown in Figure 15b for the bubble pressure of oxepan-2-one + carbon dioxide. For this system, vapor–liquid–liquid equilibrium (VLLE) can be seen at 303 K.^125^ At temperatures slightly higher than the critical point of CO_2_ (303 K), an upper critical end point signals the end of the three-phase line, and continuous behavior, from VLE at low pressure to LLE at higher pressure, can be seen (in the figure, this behavior is observed at *T* ≥ 313 K). The calculations are in very good agreement with the experimental data, although the critical pressure is overestimated in the case of the oxolan-2-one mixture (Figure 15a). The critical pressures are also slightly overestimated, but the overall agreement in terms of the VLLE (at 303 K) and the high-pressure fluid-phase equilibria (at higher temperatures) shown in Figure 15b is very good. The corresponding %AADs and AADs are detailed in Table 15. When inconsistent sets of data are found, we calculate the %AADs and AADs for the references taken together (denoted as † in the table) or taken separately (denoted as ††).

**Table 15:** Overview of the Accuracy of SAFT-γ Mie in the Calculation of Bubble Pressures *P*_bub_, Dew Pressures *P*_dew_, and Densities ρ for Mixtures of Lactones and Carbon Dioxide, Where *N*_*s*,*p*_^D^ Is the Number of Experimental Data Used in the Parameter Estimation, and *N*_*s*,*p*_^D,total^ Is the Number of Experimental Data Used to Calculate %AAD_*s*_*p* and AAD_*s*_*p* for System *s* and Property *p*^a^

system *s* (1 + 2)	*T*/K range	*P*/MPa range	*x*_1_ range	*N*_*s*,*p*_^D^	*N*_*s*,*p*_^total^	%AAD_*s*_*P*_bub_	AAD_*s*_*P*_bub_/MPa	figure	ref
oxolan-2-one + carbon dioxide	273–363	−	0.00–0.767	0	147	9.537	1.266	−	(123,124)^†^
	313–363	−	0.144–0.494	0	59	16.70	2.785	15(a)	(124)^††^
	273–313	−	0.00–0.767	0	88	4.815	0.2649	−	(123)^††^
oxepan-2-one + carbon dioxide	303–363	−	0.0199–0.770	7	106	24.03	3.190	−	(124,125)^†^
	303–343	−	0.0199–0.770	7	50	17.51	1.389	15(b)	(125)^††^
	313–363	−	0.129–0.560	0	56	29.86	4.799	−	(124)^††^
5-methyloxolan-2-one + carbon dioxide	313–373	−	0.211–0.945	0	32	8.679	0.6585	15(a)	(126)
5-ethyloxolan-2-one + carbon dioxide	303–363	−	0.0199–0.606	0	102	13.54	1.617	−	(124,125)^†^
	303–343	−	0.0199–0.606	0	55	8.733	0.7446	15(a)	(125)^††^
	323–363	−	0.140–0.513	0	47	19.16	2.637	−	(124)^††^
5-hexyloxolan-2-one + carbon dioxide	333–363	−	0.081–0.372	0	57	16.74	2.513	−	(124)
6-methyloxan-2-one + carbon dioxide	303–343	−	0.141–0.472	0	35	9.415	0.8300	−	(125)

system *s* (1 + 2)	*T*/K range	*P*/MPa range	*x*_1_ range	*N*_*s*,*p*_^D^	*N*_*s*,*p*_^total^	%AAD_*s*_*P*_dew_	AAD_*s*_*P*_dew_/MPa	figure	ref
oxolan-2-one + carbon dioxide	313–363	−	0.018–0.0379	0	63	3.916	0.5872	15(a)	(124)
oxepan-2-one + carbon dioxide	313–363	−	0.0125–0.0888	0	74	5.303	0.7419	−	(124,125)^†^
	313–343	−	0.0199–0.0888	0	22	4.903	0.8137	15(b)	(125)^††^
	313–363	−	0.0125–0.0240	0	52	5.450	0.7157	−	(124)^††^
5-methyloxolan-2-one + carbon dioxide	323–363	−	0.0140–0.0265	0	40	3.245	0.4545	−	(124)
5-ethyloxolan-2-one + carbon dioxide	313–343	−	0.0199–0.0637	0	10	5.968	0.8196	15(a)	(125)
5-hexyloxolan-2-one + carbon dioxide	323–363	−	0.0078–0.0150	0	35	6.850	0.9009	−	(124)
6-methyloxan-2-one + carbon dioxide	313–343	−	0.0282–0.114	0	20	8.166	1.092	−	(125)

system *s* (1 + 2)	*T*/K range	*P*/MPa range	*x*_1_ range	*N*_*s*,*p*_^D^	*N*_*s*,*p*_^total^	%AAD_*s*_ ρ	AAD_*s*_ ρ/(kg/m^3^)	figure	ref
oxolan-2-one + carbon dioxide	273–303	0.101	0.0416–0.571	0	16	1.0088	9.691	−	(123)

a The dagger symbols indicate that several sets of experimental data can be considered for the %ADD and AAD calculations and are taken together (†) or separately (††).

### 3.8 Overall Deviations

**Figure 16:**
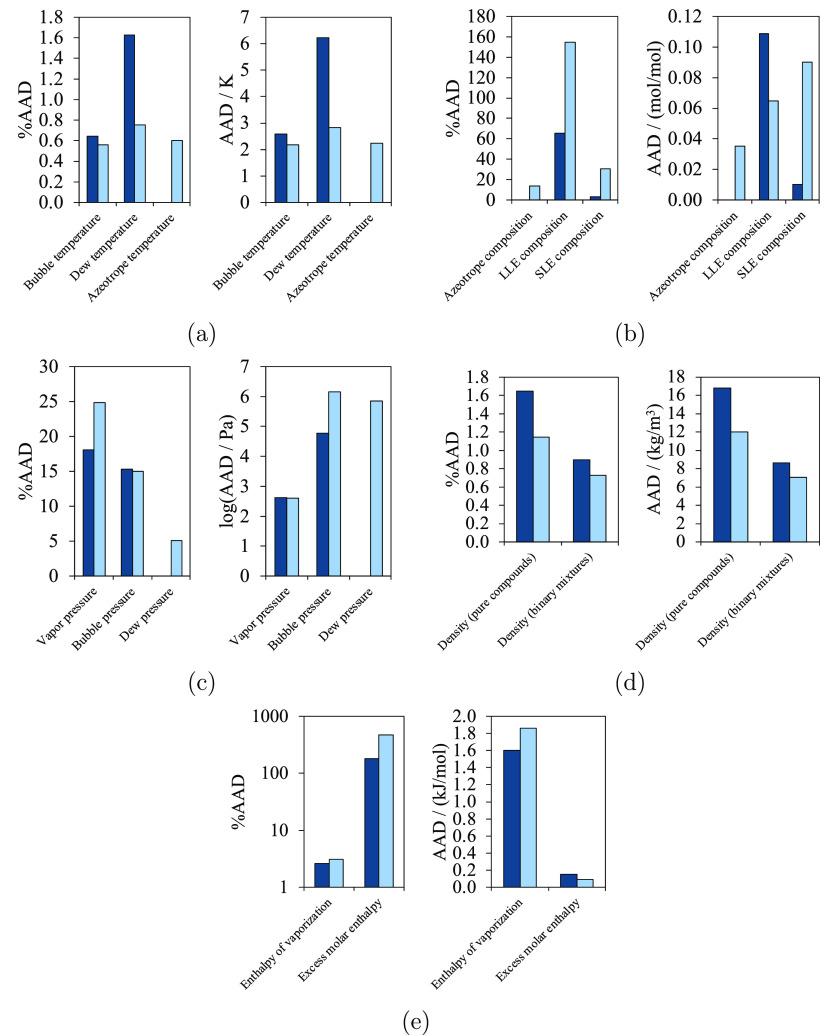
Overall %AAD and AAD for the properties considered in our current work. The results for the data used in the parameter estimation are presented in dark blue, and the results for the predictions are in pale blue. (a) Bubble, dew, and azeotrope temperatures. (b) Azeotrope, LLE, and SLE compositions. (c) Vapor pressure for pure fluids and bubble and dew pressures for mixtures. (d) Density for pure fluids and for mixtures. (e) Enthalpy of vaporization for pure fluids and excess molar enthalpy for mixtures.

As a summary of the accuracy of the calculation of the thermodynamic properties and phase equilibria obtained with the SAFT-γ Mie models presented, we collate in Table 16 and Figure 16 the overall deviations of the predicted and calculated data for each property considered. Deviations for temperature, composition, pressure, density, and enthalpy data types are presented in Figure 16 a–e, respectively. As can be seen, the values of the deviations calculated for points not used in parameter estimation (%AAD^prediction^ and AAD^prediction^) are found to be similar to those calculated for points used in parameter estimation (%AAD^estimation^ and AAD^estimation^), thereby confirming the robustness of the models. Moreover, in the case of bubble and dew temperatures (Figure 16a), as well as for densities for pure fluids and mixtures (Figure 16d), the %AAD^prediction^ values are found to be smaller than those of the %AAD^estimation^. Azeotrope temperature data are not used in parameter estimation, and we thus report only the corresponding %AAD^prediction^ and AAD^prediction^; these deviations are found to be similar to those for bubble and dew temperatures. In terms of the composition deviations, it is important to note that the %AADs for compositions in binary mixtures (i.e., *x*_1_^LLE^ and *x*_1_^sat^) depend on the choice of compound 1 as a reference. The AADs provide a better metric of performance because these do not depend on the reference compound and they can be compared with the full range of mole fraction values (i.e., from 0 to 1). We find that the AAD^estimation^ and AAD^prediction^ values of the LLE and SLE compositions are small and of similar order of magnitude (Figure 16b). Similarly, in the case of the excess molar enthalpies, the overall %AADs are rather large, while the AADs are reasonably small; this is because of the experimental data values close to zero leading to very large %AADs. In particular, it is interesting to note that %AAD^prediction^ is higher than %AAD^estimation^ for the excess molar enthalpy (470.8% and 181.8%, respectively), while the corresponding AAD^prediction^ is lower than AAD^estimation^ (92.24 J/mol and 153.7 J/mol, respectively).

**Table 16:** Overview of the Accuracy of SAFT-γ Mie Calculations for the Pure Lactones and the Binary Mixtures That Contain Lactones Considered in Our Work^a^

property *p*	*N*_*p*_^estimation^	*N*_*p*_^prediction^	%AAD^estimation^	%AAD^prediction^	AAD^estimation^	AAD^prediction^
vapor pressure	109	176	18.06	24.81	416.1 Pa	393.4 Pa
liquid density (pure compounds)	34	425	1.646	1.147	16.82 kg/m^3^	12.04 kg/m^3^
enthalpy of vaporization	110	152	2.623	3.071	1.600 kJ/mol	1.859 kJ/mol
bubble temperature	170	158	0.6427	0.5584	2.593 K	2.170 K
dew temperature	116	201	1.626	0.7560	6.217 K	2.828 K
azeotrope temperature	0	24	−	0.6022	−	2.231 K
azeotrope composition	0	36	−	13.76	−	0.03520
LLE composition	62	66	65.57	154.4	0.1088	0.06470
SLE composition	73	95	3.087	30.76	0.01025	0.09023
bubble pressure	172	619	15.30	14.97	58.50 kPa	1410 kPa
dew pressure	0	242	−	5.083	−	708.4 kPa
liquid density (binary mixtures)	220	765	0.8953	0.7271	8.618 kg/m^3^	7.063 kg/m^3^
excess molar enthalpy	322	269	181.8	470.8	0.1537 kJ/mol	0.09224 kJ/mol

a *N*_*p*_^estimation^ is the number of experimental data used in parameter estimation for property *p*. *N*_*p*_^prediction^ is the number of experimental data not used in parameter estimation (prediction only) for property *p*. We calculated the corresponding %AAD^estimation^, %AAD^prediction^, AAD^estimation^, and AAD^prediction^.

## 4 Conclusions

The thermodynamic properties and phase behavior of small saturated lactones have been modeled with the SAFT-γ Mie group-contribution approach. A total of 86 systems have been considered, which correspond to 13 pure lactones and 73 binary mixtures: 21 with saturated hydrocarbons, 20 with alcohols, 2 with ketones, 4 with esters, 15 with aromatic compounds, 5 with water, and 6 with carbon dioxide.

A new SAFT-γ Mie cCOO group has been introduced, and the relevant like cCOO–cCOO and 17 unlike group interactions have been characterized. The accuracy of the calculations has been assessed by comparison with experimental data graphically with phase diagrams over broad thermodynamic conditions and by calculating the appropriate %AADs and AADs. The overall agreement between the experimental values and the SAFT-γ Mie calculations is found to be very good for all of the mixtures and properties studied.

A number of interesting regular features in the thermodynamic properties are found from the comparison between the available experimental data and the SAFT-γ Mie calculations. The VLE for the mixtures of 5-methyloxolan-2-one and several solvents,^95,101,105,108^ including alcohols (Figures 7a and 9a), ketones (Figure 10a), esters (Figure 11a), aromatic compounds (Figure 12a), and carbon dioxide (Figure 15), do not present azeotropes. In the case of the solubility of oxepan-2-one in ketones (Figure 10b) and aromatic compounds (Figure 12b), eutectic points are found. The density of oxolan-2-one mixtures is abundantly documented,^96−98,102,106,116^ and we have presented results for mixtures with alcohols (Figures 7c and 9b), esters (Figure 11c), aromatic compounds (Figure 13a), and water (Figure 14b). The density is found to be nonideal in most of the cases considered, with a concave or convex shape as a function of mole fraction. The values found for the excess molar enthalpy, both in the literature^88,89,96,100,103,105,118^ and from the SAFT-γ Mie calculations, are generally small as can be seen for the mixtures of oxolan-2-one with hydrocarbons (Table 8), alcohols (Figure 7d), esters (Figure 11d), aromatic compounds (Figure 13b), and water (Figure 14c).

The parameters characterized in this work are transferable to other lactones, given the group-contribution nature of the SAFT-γ Mie equation of state, such that more complex molecules modeled with the cCOO group can be considered in future work. In particular, the new interaction parameters pave the way for the modeling of a wide range of compounds, for example, unsaturated lactones, ascorbic acid (vitamin C), and active pharmaceutical ingredients (e.g., simvastatin and lovastatin) of current interest.

## Data Availability

Data underlying this article can be accessed on Zenodo at DOI: 10.5281/zenodo.8268756 and used under the Creative Commons Attribution license.
